# Genetic forms of primary progressive aphasia within the GENetic Frontotemporal dementia Initiative (GENFI) cohort: comparison with sporadic primary progressive aphasia

**DOI:** 10.1093/braincomms/fcad036

**Published:** 2023-02-17

**Authors:** Kiran Samra, Amy M MacDougall, Arabella Bouzigues, Martina Bocchetta, David M Cash, Caroline V Greaves, Rhian S Convery, Chris Hardy, John C van Swieten, Harro Seelaar, Lize C Jiskoot, Fermin Moreno, Raquel Sanchez-Valle, Robert Laforce, Caroline Graff, Mario Masellis, Maria Carmela Tartaglia, James B Rowe, Barbara Borroni, Elizabeth Finger, Matthis Synofzik, Daniela Galimberti, Rik Vandenberghe, Alexandre de Mendonça, Chris R Butler, Alexander Gerhard, Simon Ducharme, Isabelle Le Ber, Isabel Santana, Florence Pasquier, Johannes Levin, Markus Otto, Sandro Sorbi, Jason D Warren, Jonathan D Rohrer, Lucy L Russell, GENetic Frontotemporal dementia Initiative (GENFI), Sónia Afonso, Maria Rosario Almeida, Sarah Anderl-Straub, Christin Andersson, Anna Antonell, Silvana Archetti, Andrea Arighi, Mircea Balasa, Myriam Barandiaran, Nuria Bargalló, Robart Bartha, Benjamin Bender, Alberto Benussi, Maxime Bertoux, Anne Bertrand, Valentina Bessi, Sandra Black, Sergi Borrego-Ecija, Jose Bras, Alexis Brice, Rose Bruffaerts, Agnès Camuzat, Marta Cañada, Valentina Cantoni, Paola Caroppo, Miguel Castelo-Branco, Olivier Colliot, Thomas Cope, Vincent Deramecourt, María de Arriba, Giuseppe Di Fede, Alina Díez, Diana Duro, Chiara Fenoglio, Camilla Ferrari, Catarina B Ferreira, Nick Fox, Morris Freedman, Giorgio Fumagalli, Aurélie Funkiewiez, Institut du Cerveau, Alazne Gabilondo, Roberto Gasparotti, Serge Gauthier, Stefano Gazzina, Giorgio Giaccone, Ana Gorostidi, Rita Guerreiro, Carolin Heller, Tobias Hoegen, Begoña Indakoetxea, Vesna Jelic, Hans-Otto Karnath, Ron Keren, Gregory Kuchcinski, Tobias Langheinrich, Thibaud Lebouvier, Maria João Leitão, Albert Lladó, Gemma Lombardi, Sandra Loosli, Carolina Maruta, Simon Mead, Lieke Meeter, Gabriel Miltenberger, Rick van Minkelen, Sara Mitchell, Katrina Moore, Benedetta Nacmias, Annabel Nelson, Linn Öijerstedt, Jaume Olives, Sebastien Ourselin, Alessandro Padovani, Jessica Panman, Janne M Papma, Yolande Pijnenburg, Cristina Polito, Enrico Premi, Sara Prioni, Catharina Prix, Rosa Rademakers, Veronica Redaelli, Daisy Rinaldi, Institut du Cerveau, Tim Rittman, Ekaterina Rogaeva, Adeline Rollin, Pedro Rosa-Neto, Giacomina Rossi, Martin Rossor, Beatriz Santiago, Dario Saracino, Sabrina Sayah, Elio Scarpini, Sonja Schönecker, Elisa Semler, Rachelle Shafei, Christen Shoesmith, Imogen Swift, Miguel Tábuas-Pereira, Mikel Tainta, Ricardo Taipa, David Tang-Wai, David L Thomas, Paul Thompson, Hakan Thonberg, Carolyn Timberlake, Pietro Tiraboschi, Emily Todd, Philip Van Damme, Mathieu Vandenbulcke, Michele Veldsman, Ana Verdelho, Jorge Villanua, Carlo Wilke, Ione Woollacott, Elisabeth Wlasich, Henrik Zetterberg, Miren Zulaica

**Affiliations:** Dementia Research Centre, Department of Neurodegenerative Disease, UCL Queen Square Institute of Neurology, London, UK; Department of Medical Statistics, London School of Hygiene and Tropical Medicine, London, UK; Dementia Research Centre, Department of Neurodegenerative Disease, UCL Queen Square Institute of Neurology, London, UK; Dementia Research Centre, Department of Neurodegenerative Disease, UCL Queen Square Institute of Neurology, London, UK; Dementia Research Centre, Department of Neurodegenerative Disease, UCL Queen Square Institute of Neurology, London, UK; Dementia Research Centre, Department of Neurodegenerative Disease, UCL Queen Square Institute of Neurology, London, UK; Dementia Research Centre, Department of Neurodegenerative Disease, UCL Queen Square Institute of Neurology, London, UK; Dementia Research Centre, Department of Neurodegenerative Disease, UCL Queen Square Institute of Neurology, London, UK; Department of Neurology, Erasmus Medical Centre, Rotterdam, Netherlands; Department of Neurology, Erasmus Medical Centre, Rotterdam, Netherlands; Department of Neurology, Erasmus Medical Centre, Rotterdam, Netherlands; Cognitive Disorders Unit, Department of Neurology, Donostia Universitary Hospital, San Sebastian, Spain; Neuroscience Area, Biodonostia Health Research Institute, San Sebastian, Gipuzkoa, Spain; Alzheimer’s disease and Other Cognitive Disorders Unit, Neurology Service, Hospital Clínic, Institut d’Investigacións Biomèdiques August Pi I Sunyer, University of Barcelona, Barcelona, Spain; Clinique Interdisciplinaire de Mémoire, Département des Sciences Neurologiques, CHU de Québec, and Faculté de Médecine, Université Laval, Québec City, Canada; Center for Alzheimer Research, Division of Neurogeriatrics, Department of Neurobiology, Care Sciences and Society, Bioclinicum, Karolinska Institutet, Solna, Sweden; Unit for Hereditary Dementias, Theme Aging, Karolinska University Hospital, Solna, Sweden; Sunnybrook Health Sciences Centre, Sunnybrook Research Institute, University of Toronto, Toronto, Canada; Tanz Centre for Research in Neurodegenerative Diseases, University of Toronto, Toronto, ON, Canada; Department of Clinical Neurosciences, University of Cambridge, Cambridge, UK; Neurology Unit, Department of Clinical and Experimental Sciences, University of Brescia, Brescia, Italy; Department of Clinical Neurological Sciences, University of Western Ontario, London, ON, Canada; Department of Neurodegenerative Diseases, Hertie-Institute for Clinical Brain Research and Center of Neurology, University of Tübingen, Tübingen, Germany; Centre for Neurodegenerative Diseases (DZNE), Tübingen, Germany; Fondazione Ca’ Granda, IRCCS Ospedale Policlinico, Milan, Italy; University of Milan, Centro Dino Ferrari, Milan, Italy; Laboratory for Cognitive Neurology, Department of Neurosciences, KU Leuven, Leuven, Belgium; Neurology Service, University Hospitals Leuven, Leuven, Belgium; Leuven Brain Institute, KU Leuven, Leuven, Belgium; Laboratory of Neurosciences, Institute of Molecular Medicine, Faculty of Medicine, University of Lisbon, Lisbon, Portugal; Nuffield Department of Clinical Neurosciences, Medical Sciences Division, University of Oxford, Oxford, UK; Department of Brain Sciences, Imperial College London, London, UK; Division of Neuroscience and Experimental Psychology, Wolfson Molecular Imaging Centre, University of Manchester, Manchester, UK; Departments of Geriatric Medicine and Nuclear Medicine, University of Duisburg-Essen, Germany; Department of Psychiatry, McGill University Health Centre, McGill University, Montreal, Québec, Canada; McConnell Brain Imaging Centre, Montreal Neurological Institute, McGill University, Montreal, Québec, Canada; Sorbonne Université, Paris Brain Institute – Institut du Cerveau – ICM, Inserm U1127, CNRS UMR 7225, AP-HP - Hôpital Pitié-Salpêtrière, Paris, France; Centre de référence des démences rares ou précoces, IM2A, Département de Neurologie, AP-HP - Hôpital Pitié-Salpêtrière, Paris, France; Département de Neurologie, AP-HP - Hôpital Pitié-Salpêtrière, Paris, France; University Hospital of Coimbra (HUC), Neurology Service, Faculty of Medicine, University of Coimbra, Coimbra, Portugal; Center for Neuroscience and Cell Biology, Faculty of Medicine, University of Coimbra, Coimbra, Portugal; Univ Lille, Inserm 1172, Lille, France; Inserm 1172, Lille, France; CHU, CNR-MAJ, Labex Distalz, LiCEND Lille, France; Department of Neurology, Ludwig-Maximilians Universität München, Munich, Germany; German Centre for Neurodegenerative Diseases (DZNE), Munich, Germany; Munich Cluster of Systems Neurology (SyNergy), Munich, Germany; Department of Neurology, University of Ulm, Ulm, Germany; Department of Neurofarba, University of Florence, Florence, Italy; IRCCS Fondazione Don Carlo Gnocchi, Florence, Italy; Dementia Research Centre, Department of Neurodegenerative Disease, UCL Queen Square Institute of Neurology, London, UK; Dementia Research Centre, Department of Neurodegenerative Disease, UCL Queen Square Institute of Neurology, London, UK; Dementia Research Centre, Department of Neurodegenerative Disease, UCL Queen Square Institute of Neurology, London, UK

**Keywords:** primary progressive aphasia, *GRN*, *c9orf72*, *MAPT*

## Abstract

Primary progressive aphasia is most commonly a sporadic disorder, but in some cases, it can be genetic. This study aimed to understand the clinical, cognitive and imaging phenotype of the genetic forms of primary progressive aphasia in comparison to the canonical nonfluent, semantic and logopenic subtypes seen in sporadic disease. Participants with genetic primary progressive aphasia were recruited from the international multicentre GENetic Frontotemporal dementia Initiative study and compared with healthy controls as well as a cohort of people with sporadic primary progressive aphasia. Symptoms were assessed using the GENetic Frontotemporal dementia Initiative language, behavioural, neuropsychiatric and motor scales. Participants also underwent a cognitive assessment and 3 T volumetric T1-weighted MRI. One *C9orf72* (2%), 1 *MAPT* (6%) and 17 *GRN* (44%) symptomatic mutation carriers had a diagnosis of primary progressive aphasia. In the *GRN* cohort, 47% had a diagnosis of nonfluent variant primary progressive aphasia, and 53% had a primary progressive aphasia syndrome that did not fit diagnostic criteria for any of the three subtypes, called primary progressive aphasia-not otherwise specified here. The phenotype of the genetic nonfluent variant primary progressive aphasia group largely overlapped with that of sporadic nonfluent variant primary progressive aphasia, although the presence of an associated atypical parkinsonian syndrome was characteristic of sporadic and not genetic disease. The primary progressive aphasia -not otherwise specified group however was distinct from the sporadic subtypes with impaired grammar/syntax in the presence of relatively intact articulation, alongside other linguistic deficits. The pattern of atrophy seen on MRI in the genetic nonfluent variant primary progressive aphasia group overlapped with that of the sporadic nonfluent variant primary progressive aphasia cohort, although with more posterior cortical involvement, whilst the primary progressive aphasia-not otherwise specified group was strikingly asymmetrical with involvement particularly of the insula and dorsolateral prefrontal cortex but also atrophy of the orbitofrontal cortex and the medial temporal lobes. Whilst there are overlapping symptoms between genetic and sporadic primary progressive aphasia syndromes, there are also distinct features. Future iterations of the primary progressive aphasia consensus criteria should encompass such information with further research needed to understand the earliest features of these disorders, particularly during the prodromal period of genetic disease.

## Teaser

Samra *et al*. report that progranulin mutations are the commonest genetic cause of primary progressive aphasia. Two subtypes are seen: a nonfluent variant (similar in phenotype to its sporadic counterpart) and a ‘not otherwise specified’ variant (with unique linguistic features). Revised criteria for primary progressive aphasia should consider genetic phenotypes.

## Graphical Abstract

**Graphical abstract:**
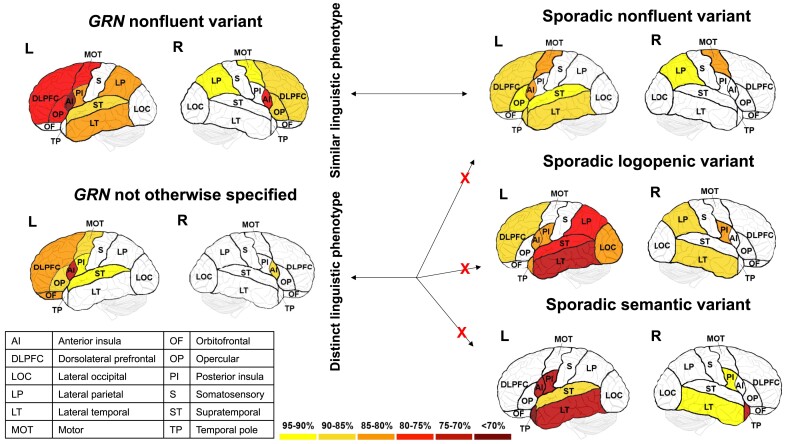

## Introduction

Frontotemporal dementia (FTD) is a neurodegenerative disorder characterised by progressive changes in behaviour, language and motor function.^1^ Although the most common presenting symptom is a change in behaviour (behavioural variant FTD, bvFTD), many people’s initial difficulties are with language, a condition termed primary progressive aphasia (PPA). Three subtypes of PPA are described:^2^ semantic variant PPA (svPPA) is characterised by anomia and impaired word comprehension with later non-verbal semantic impairment; nonfluent variant PPA (nfvPPA) presents with apraxia of speech and/or impaired grammar; and logopenic variant PPA (lvPPA) is associated with word retrieval difficulties as well as impaired sentence repetition and comprehension. However, a number of studies have now shown that a substantial minority of people with PPA do not fit into any of the three main subtypes, a disorder often called mixed PPA or PPA-not otherwise specified (PPA-NOS).^3,4^

Whilst the majority of people with PPA have a sporadic disease, cases of familial PPA have been described for many years.^5,6^ Overall, between a third and a half of individuals with FTD have a familial disorder^7^ with mutations in progranulin (*GRN*), microtubule-associated protein tau (*MAPT*) and chromosome 9 open reading frame 72 (*C9orf72*) being the commonest genetic causes. However, the heritability of PPA is less than that of bvFTD, with only 30% of nfvPPA, 22% of svPPA and 20% of lvPPA being found to have a family history (of at least one first-degree relative with dementia) in one previous study.^7^*GRN* mutations have previously been described as the most common genetic cause of PPA,^8^ with fewer reports of PPA in the other genetic groups.^9^ The phenotype of such familial PPA cases has been poorly studied but has been noted, particularly in those with *GRN* mutations, as often not easily characterized into one of the three canonical subtypes^8,10–15^, i.e. to fall into the PPA-NOS group.

In the sporadic forms of PPA, language impairment can be the only feature for a number of years into the illness.^16,17^ However, other cognitive, behavioural, neuropsychiatric and motor features can be associated with each of the different variants with increasing disease progression. People with svPPA may develop non-verbal semantic impairment and behavioural symptoms,^18^ whilst people with nfvPPA can develop dyscalculia and limb apraxia as well as parkinsonism, including features consistent with either a corticobasal syndrome (CBS) or progressive supranuclear palsy (PSP).^19,20^ In contrast, people with lvPPA often develop episodic memory and posterior cortical deficits, in line with the disorder being commonly an atypical form of Alzheimer’s disease (AD).^2^ As with the linguistic phenotype, little is known about the non-language features of the genetic forms of PPA.

With these findings in mind, we therefore aimed to understand the linguistic and non-linguistic phenotype of the genetic forms of PPA within the Genetic Frontotemporal dementia Initiative (GENFI) cohort, by investigating the cognitive, behavioural, neuropsychiatric and motor features of the condition, in comparison with healthy controls as well as sporadic forms of PPA.

## Materials and methods

### Participants

Participants were recruited from the fifth data freeze of the GENFI study between 20 January 2012 and 30 May 2019, including sites in the UK, Canada, France, Belgium, Germany, Italy, Netherlands, Portugal, Spain and Sweden. All aspects of the study were approved by local ethics committees and written informed consent obtained from all participants.

Participants underwent a standardised clinical assessment including a clinical history and neurological examination, neuropsychometric assessment, the Mini-Mental State Examination (MMSE) and the CDR® plus NACC FTLD.^21^ The CDR® plus NACC FTLD was used to classify mutation carriers as asymptomatic (global score of 0), prodromal (score 0.5) or symptomatic (score ≥1). Initially, we reviewed all symptomatic mutation carriers recruited to the study and excluded those severely affected (CDR® plus NACC FTLD score of 3, i.e. only included those with a score of 1 or 2). From this remaining group of 103 participants, there were 39 *GRN*, 46 *C9orf72* and 18 *MAPT* mutation carriers (Supplementary Fig. 1). A diagnosis of PPA was made in the study by clinician assessment according to the international consensus PPA criteria.^22^ An overall PPA diagnosis was made and then, if possible, a subtype (svPPA, nfvPPA or lvPPA) was allocated to each participant. If participants did not fit specific criteria for one of the subtypes, they were diagnosed as PPA-NOS.

An initial comparison group was generated from the GENFI healthy controls (i.e. non-mutation carriers) including those who were matched on age, sex and years of education—this formed a control group of 50 participants (all with CDR® plus NACC FTLD of 0 or 0.5). A second set of comparison groups were generated from the UCL Longitudinal Investigation of Frontotemporal Dementia (LIFTD) study of sporadic FTD (i.e. all cases in the study tested negative for FTD-causing mutations), including participants with a PPA diagnosis and a CDR® plus NACC FTLD global score of 1 or 2. In total 45 people with sporadic PPA were included: 19 with svPPA, 16 with nfvPPA and 10 with lvPPA (Supplementary Fig. 1). Demographics are shown in Table 1.

**Table 1:** Demographics, clinical and linguistic symptom data for the genetic and sporadic PPA subgroups as well as healthy controls

	Controls	Genetic PPA		Sporadic PPA		
		*GRN*-NFV	*GRN*-NOS	LV	NFV	SV
Number of participants	50	8	9	10	16	19
% Male	34	50	33	**70**	**63**	**63**
Age (years)	65.4 (4.5)	67.4 (7.9)	63.5 (8.2)	68.7 (6.4)	67.3 (6.1)	64.0 (6.8)
Education (years)	13.2 (2.9)	13.6 (2.7)	12.7 (3.9)	14.4 (2.4)	14.0 (2.3)	14.4 (3.1)
MMSE	29.0 (1.4)	**22.4** (**6.1)**	**20.9** (**9.8)**	**19.1** (**5.5)**	**22.4** (**8.2)**	**23.0** (**7.8)**
CDR® plus NACC FTLD Global score	0.1 (0.2)	**1.4** (**0.2)**	**1.4** (**0.5)**	**1.4** (**0.5)**	**1.8** (**0.5)**	**1.6** (**0.5)**
CDR® plus NACC FTLD Sum of boxes	0.2 (0.4)	**6.0** (**3.7)**	**6.6** (**2.9)**	**6.8** (**2.7)**	**7.3** (**3.1)**	**8.0** (**3.7)**
Progressive aphasia Severity Scale Sum of Boxes	0.1 (0.2)	**16.1** (**5.9)**	**12.3** (**6.0)**	**11.9** (**3.3)**	**15.8** (**5.7)**	**10.4** (**4.0)**
Impaired articulation	0.0 (0.0)	**1.9** (**1.0)**	0.4 (0.7)	**0.5** (**0.4)**	**2.2** (**0.9)**	0.1 (0.2)
Decreased fluency	0.0 (0.0)	**2.4** (**0.5)**	**1.8** (**0.9)**	**1.3** (**0.7)**	**2.4** (**0.6)**	**0.6** (**0.6)**
Impaired grammar/syntax	0.0 (0.0)	**2.1** (**0.6)**	**1.6** (**1.0)**	**0.6** (**0.6)**	**1.7** (**1.0)**	**0.2** (**0.4)**
Impaired word retrieval	0.0 (0.1)	**1.8** (**0.9)**	**1.8** (**0.9)**	**2.0** (**0.5)**	**1.6** (**1.0)**	**1.8** (**0.5)**
Impaired speech repetition	0.0 (0.0)	**1.6** (**1.1)**	**1.3** (**1.1)**	**1.6** (**0.7)**	**1.4** (**1.1)**	**0.3** (**0.6)**
Impaired sentence comprehension	0.0 (0.0)	**0.8** (**0.7)**	**1.6** (**0.8)**	**1.2** (**0.5)**	**1.0** (**1.0)**	**1.4** (**0.9)**
Impaired single word comprehension	0.0 (0.1)	0.4 (0.7)	**0.8** (**1.0)**	**1.1** (**0.6)**	**0.6** (**0.8)**	**1.9** (**0.6)**
Dyslexia	0.0 (0.0)	**1.0** (**0.7)**	**0.6** (**0.7)**	**1.2** (**0.8)**	**1.0** (**0.9)**	**1.4** (**1.0)**
Dysgraphia	0.0 (0.1)	**2.1** (**0.9)**	**0.8** (**0.6)**	**0.9** (**0.5)**	**1.5** (**1.1)**	**1.0** (**0.9)**
Impaired functional communication	0.0 (0.0)	**2.3** (**0.7)**	**1.5** (**0.8)**	**1.7** (**0.5)**	**2.3** (**0.5)**	**1.6** (**0.6)**

Age, education, Mini-Mental State Examination (MMSE) and clinical rating scale scores are shown as mean (standard deviation).

*GRN*-NFV, nonfluent variant PPA due to progranulin mutation; *GRN*-NOS, not otherwise specified PPA due to progranulin mutation; LV, logopenic variant PPA; NFV, nonfluent variant PPA; SV, semantic variant PPA.

Bold items are significantly different to controls.

### Linguistic symptoms

Language was assessed by a clinician using the GENFI linguistic symptom scale, which is based on the Progressive Aphasia Severity Scale (PASS).^23^ This contains 10 language symptoms scored as per a Clinical Dementia Rating (CDR) scale, i.e. 0 = asymptomatic, 0.5 = questionable/very mild, 1 = mild, 2 = moderate and 3 = severe: impaired articulation, decreased fluency, impaired grammar/syntax, impaired word retrieval, impaired speech repetition, impaired sentence comprehension, impaired single word comprehension, dyslexia, dysgraphia and impaired functional communication.

### Non-linguistic (behavioural, neuropsychiatric, and motor) symptoms

Behavioural, neuropsychiatric and motor symptoms were assessed using the standardised GENFI clinical questionnaire (which uses the CDR scale of 0–3). This includes seven behavioural symptoms: disinhibition, apathy, loss of sympathy/empathy, ritualistic/compulsive behaviour, hyperorality and appetite changes, poor response to social/emotional cues and inappropriate trusting behaviour. Fourteen neuropsychiatric symptoms were assessed: visual, auditory and tactile hallucinations, delusions, depression, anxiety, irritability/lability, agitation/aggression, euphoria/elation, aberrant motor behaviour, hypersexuality, hyperreligiosity, impaired sleep and altered sense of humour. Lastly, eight motor symptoms were enquired about: dysarthria, dysphagia, tremor, slowness, weakness, gait disorder, falls and functional difficulties using hands.

### Cognitive assessment

Within the GENFI neuropsychology battery, the 30-item version of the Boston Naming Test^24,25^ (BNT) and the modified Camel and Cactus Test^26^ (mCCT) were the linguistic measures used. The rest of the GENFI neuropsychology battery includes tests of attention and executive function including the Trail Making Test Parts A and B (TMTA and TMTB), D-KEFS Color-Word Inference Test (CWIT), WAIS-R Digit Symbol test, WMS-R Digit Span Forwards (DSF) and Backwards (DSB) and category fluency (animals) as well as tests of visuospatial skills (WASI Block Design), episodic memory (Free and Cued Selective Reminding Test, FCSRT) and social cognition (mini-Social Cognition and Emotion Assessment, mini-SEA, which includes a Faux Pas test of theory of mind and a Facial Emotion Recognition Test). The LIFTD cohort underwent a subset of these tests, with none of the participants having undergone the FCSRT or mini-SEA.

### Neuroimaging analysis

Participants underwent a 3T volumetric T1-weighted MRI scan as per the harmonized GENFI protocol.^27^ The majority of participants had a scan of sufficient quality to be analysed (42 on a Siemens Prisma, 31 on a Siemens Trio, 6 on a Siemens Skyra, and 24 on a Philips Achieva): 45 of 50 controls, 14 of 17 genetic PPA, 9 of 10 lvPPA, all 16 nfvPPA and all 19 svPPA participants (see Supplementary Table 4). Those without scans had either not been scanned due to contraindications or had a poor-quality scan due to movement or other artefacts.

Volumetric MRI scans were first bias field corrected and whole brain parcellated using the geodesic information flow (GIF) algorithm,^28^ which is based on atlas propagation and label fusion. We combined regions of interest to calculate grey matter volumes of the cortex for 15 regions: orbitofrontal, dorsolateral (DLPFC) and ventromedial prefrontal (VMPFC), motor, opercular, anterior and posterior insula, temporal pole, lateral and medial temporal, supratemporal, anterior and posterior cingulate, sensory, medial and lateral parietal, and medial and lateral occipital cortex. Volumes for subcortical regions were also calculated including the amygdala, hippocampus, nucleus accumbens, caudate, globus pallidus, putamen, thalamus, together with the cerebellum. All measures were expressed as a percentage of total intracranial volume (TIV) computed with SPM12 v6470 (^29^ Statistical Parametric Mapping, Wellcome Trust Centre for Neuroimaging, London, UK) running under Matlab R2014b (Math Works, Natick, MA, USA).^30^

### Statistical analysis

All statistical analyses were performed using Stata/MP 16.1. Statistical tests of normality were performed using the Shapiro–Wilk test. Demographics were compared between groups using either linear regression (age and education) or a chi-squared test (sex). Linear regressions adjusting for age and sex were used to analyse the MMSE, CDR® plus NACC FTLD and PASS scores. Both linguistic and non-linguistic symptoms were compared in each disease group versus controls using linear regressions adjusting for age and sex and 95% bias-corrected bootstrapped confidence intervals with 2000 repetitions (as there was minimal variation from 0 in severity scores for the control group). Comparison of these symptoms between groups used an ordinal logistic regression adjusting for age and sex. The neuropsychological assessments and cortical and subcortical volumes were compared using linear regression models adjusting for age and sex, as well as scanner type for the imaging analysis; 95% bias-corrected bootstrapped confidence intervals with 2000 repetitions were used if data were not normally distributed.

### Ethics approval and consent to participate

All GENFI sites had local ethical approval for the study, and all participants gave written informed consent.

### Data availability

The datasets used and/or analysed during the current study are available from the corresponding author on reasonable request.

## Results

### Frequency of PPA in the GENFI cohort

In total 1 *C9orf72* (2%), 1 *MAPT* (6%) and 17 *GRN* (44%) mutation carriers from within the total mild to moderate symptomatic mutation carrier population had a PPA diagnosis. On assessment of their specific subtype, the *C9orf72* mutation carrier had a diagnosis of nfvPPA, the *MAPT* mutation carrier had a diagnosis of svPPA, and the *GRN* mutation carriers split into eight with a diagnosis of nfvPPA (termed *GRN*-NFV from here) and nine who did not fit diagnostic criteria for any specific PPA variant, i.e. PPA-NOS (termed *GRN*-NOS from here). Due to the small numbers in the *C9orf72* and *MAPT* mutation groups, we focused further analyses on the *GRN*-PPA group—details of the non-*GRN* cases are shown in Supplementary Table 1.

### Demographics

No significant differences were seen between the *GRN*-PPA group and controls in age, sex or education. However, the sporadic lvPPA, nfvPPA and svPPA groups had a significantly higher percentage of males than the control group (*Chi*^2^ = 4.50, *P* = 0.034; *Chi*^2^ = 4.07, *P* = 0.044; *Chi*^2^ = 4.80, *P* = 0.028, respectively). The sporadic lvPPA cohort was older than the svPPA group (*P* = 0.045), but there were no other differences in age and no significant differences in education between the groups.

### Disease severity

CDR® plus NACC FTLD sum of boxes and MMSE scores were all significantly different to controls in each of the disease groups, but there were no significant differences between the PPA groups.

#### Linguistic symptoms

**Figure 1:**
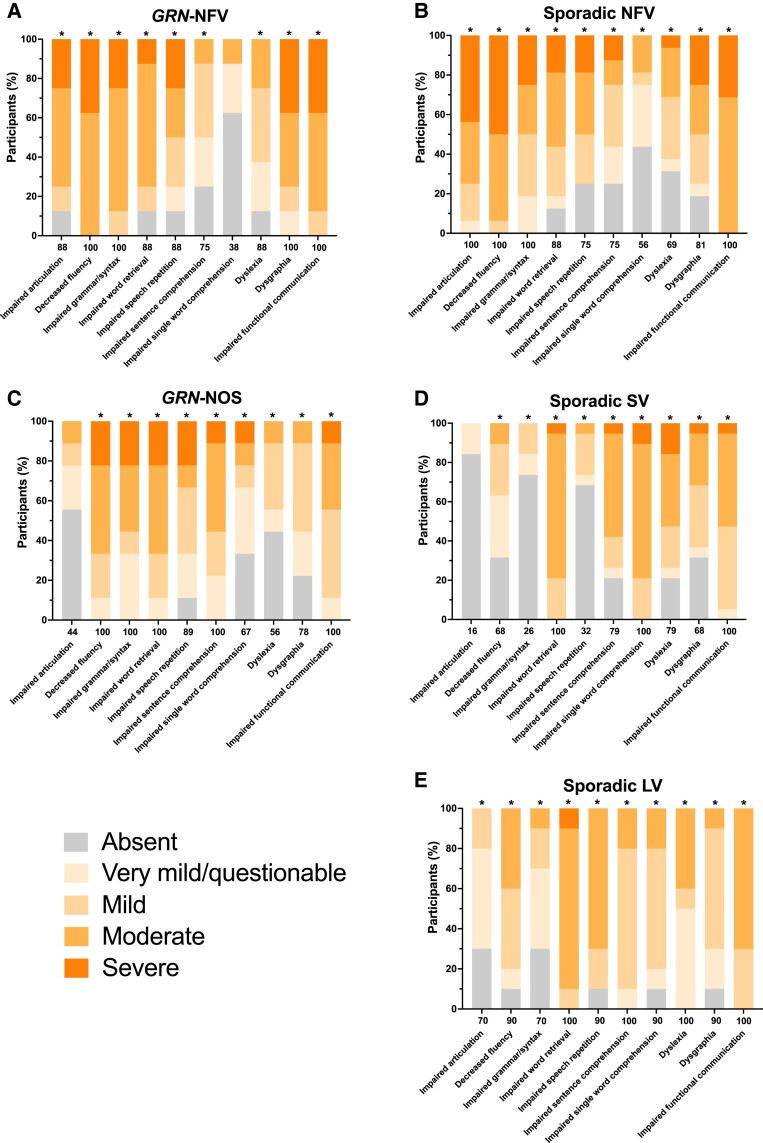
**Linguistic symptoms showing the percentage of participants in each of the PPA groups who score 0 = absent, 0.5 = very mild/questionable, 1 = mild, 2 = moderate or 3 = severe for each symptom. A**, *GRN*-NFV; **B**, Sporadic NFV; **C**, *GRN*-NOS; **D**, Sporadic SV; **E**, Sporadic LV. Values along the x-axis represent the frequency (%) with which the symptom is present in any severity category (0.5–3). An asterisk above the bar indicates that the symptom severity is significantly greater than controls. Linear regressions adjusting for age and sex were used to compare groups for each symptom: impaired articulation [Wald chi^2^(7) = 146.7, *P* < 0.001; *R*^2^ = 0.761]; decreased fluency [Wald chi^2^(7) = 536.0, *P* < 0.001; *R*^2^ = 0.811]; impaired grammar/syntax [Wald chi^2^(7) = 176.1, *P* < 0.001; *R*^2^ = 0.682]; impaired word retrieval [Wald chi^2^(7) = 538.8, *P* < 0.001; *R*^2^ = 0.744]; impaired speech repetition [Wald chi^2^(7) = 116.3, *P* < 0.001; *R*^2^ = 0.535]; impaired sentence comprehension [Wald chi^2^(7) = 155.4, *P* < 0.001; *R*^2^ = 0.521]; impaired single word comprehension [Wald chi^2^(7) = 250.2, *P* < 0.001; *R*^2^ = 0.638]; dyslexia [Wald chi^2^(7) = 111.3, *P* < 0.001; *R*^2^ = 0.470]; dysgraphia [Wald chi^2^(7) = 146.2, *P* < 0.001; *R*^2^ = 0.537]; impaired functional communication [Wald chi^2^(7) = 688.6, *P* < 0.001; *R*^2^ = 0.830]. Abbreviations: *GRN*-NFV, nonfluent variant PPA due to progranulin mutation; *GRN*-NOS, not otherwise specified PPA due to progranulin mutation; LV, logopenic variant PPA; NFV, nonfluent variant PPA; SV, semantic variant PPA.

The majority of controls showed no symptoms of language impairment, with only 6% having impaired word retrieval, 2% having impaired single word comprehension and 2% with dysgraphia (all at a 0.5 very mild/questionable severity). All other linguistic symptoms did not occur in controls. In contrast, all of the linguistic symptoms were present within each of the PPA subtypes in at least some of the patients in each group (Fig. 1, Table 1).

As expected, the most frequent and severe symptoms in the sporadic forms of PPA were decreased fluency [mean (SD) 2.4 (0.6)], impaired articulation [2.2 (0.9)] and impaired grammar/syntax [1.7 (1.0)] in the nfvPPA group, impaired single word comprehension [1.9 (0.6)] and impaired word retrieval [1.8 (0.5)] in the svPPA group, and impaired word retrieval [2.0 (0.5)], impaired speech repetition [1.6 (0.7)], impaired sentence comprehension [1.2 (0.5)] and dyslexia [1.2 (0.8)] in the lvPPA group. Apart from impaired speech repetition in lvPPA (occurring in 90%), these symptoms occurred in 100% of patients with these PPA subtypes. The least frequent and severe symptoms in the sporadic forms of PPA were also as expected: impaired single word comprehension in nfvPPA [0.6 (0.8), 56%] and both impaired articulation [0.1 (0.2), 16%; 0.5 (0.4), 70%] and impaired grammar/syntax [0.2 (0.4), 26%; 0.6 (0.6), 70%] in the svPPA and lvPPA groups. Nonetheless, in comparing severity of symptoms versus controls, all were significant except impaired articulation for svPPA (*P* = 0.144).

The genetic nfvPPA group (*GRN*-NFV) had an overlapping pattern of symptoms to the sporadic nfvPPA group (Fig. 1A and B, Table 1): the most frequent and severe symptoms were decreased fluency [2.4 (0.5), 100%], impaired grammar/syntax [2.1 (0.6), 100%] and impaired articulation [1.9 (1.0), 88%] as with sporadic nfvPPA, with the addition in the *GRN*-NFV group of dysgraphia [2.1 (0.9), 100%], whilst the least frequent and severe symptom was impaired single word comprehension [0.4 (0.7), 38%]. This latter symptom being the only one not significantly different in severity to controls (*P* = 0.186).

The genetic PPA-NOS group (*GRN*-NOS) had a different pattern to any of the sporadic PPA groups or the *GRN*-NFV group. The most frequent and severe symptoms were decreased fluency [1.8 (0.9), 100%], impaired word retrieval [1.8 (0.9), 100%], impaired grammar/syntax [1.6 (1.0), 100%] and impaired sentence comprehension [1.6 (0.8), 100%], whilst the least frequent and severe symptom was impaired articulation [0.4 (0.7), 44%], this latter symptom being the only one not significantly different in severity to controls (*P* = 0.063). Of note, impaired speech repetition occurred in 89% [1.3 (1.1)], and impaired single word comprehension occurred in 67% [0.8 (1.0)] in this group.

Results comparing each linguistic symptom across the PPA groups are shown in Supplementary Table 2. Characteristic between-group differences were seen in sporadic PPA, i.e. more severe impairments of articulation and grammar/syntax in nfvPPA, single word comprehension in svPPA and speech repetition in lvPPA. Within the genetic PPA groups, *GRN*-NFV had more severely impaired articulation (*P* = 0.001) and dysgraphia (*P* = 0.002) than the *GRN*-NOS group, whilst the *GRN*-NOS group had more severely impaired sentence comprehension than *GRN*-NFV (*P* = 0.026).

#### Non-linguistic symptoms

**Figure 2:**
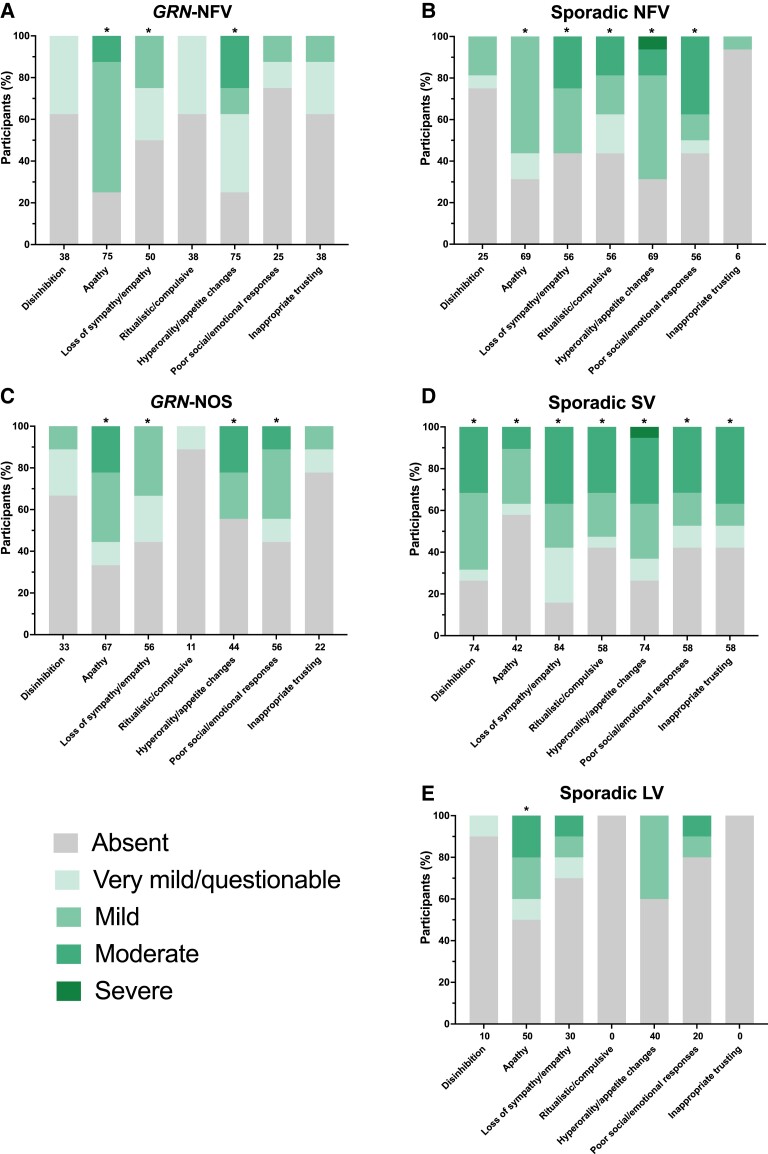
**Behaviour symptoms showing the percentage of participants in each of the PPA groups who score 0 = absent, 0.5 = very mild/questionable, 1 = mild, 2 = moderate, or 3 = severe for each symptom. A**, *GRN*-NFV; **B**, Sporadic NFV; **C**, *GRN*-NOS; **D**, Sporadic SV; **E**, Sporadic LV. Values along the x-axis represent the frequency (%) with which the symptom is present in any severity category (0.5–3). An asterisk above the bar indicates that the symptom severity is significantly greater than controls. Linear regressions adjusting for age and sex were used to compare groups for each symptom: disinhibition [Wald chi^2^(7) = 44.9, *P* < 0.001; *R*^2^ = 0.494]; apathy [Wald chi^2^(7) = 70.6, *P* < 0.001; *R*^2^ = 0.348]; loss of sympathy/empathy [Wald chi^2^(7) = 73.7, *P* < 0.001; *R*^2^ = 0.436]; ritualistic/compulsive [Wald chi^2^(7) = 35.4, *P* < 0.001; *R*^2^ = 0.358]; hyperorality/appetite changes [Wald chi^2^(7) = 66.0, *P* < 0.001; *R*^2^ = 0.377]; poor social/emotional responses [Wald chi^2^(7) = 50.0, *P* < 0.001; *R*^2^ = 0.359]; inappropriate trusting [Wald chi^2^(7) = 28.5, *P* < 0.001; *R*^2^ = 0.400]. Abbreviations: *GRN*-NFV, nonfluent variant PPA due to progranulin mutation; *GRN*-NOS, not otherwise specified PPA due to progranulin mutation; LV, logopenic variant PPA; NFV, nonfluent variant PPA; SV, semantic variant PPA.

Behavioural symptoms occurred commonly in patients in each group compared with only 10% of controls: svPPA (95% of patients), sporadic nfvPPA (94%), genetic nfvPPA (88%), genetic PPA-NOS (78%) and lvPPA (70%) (Fig. 2A–E).

In svPPA the most frequent and severe symptoms were loss of sympathy/empathy [84%, 1.1 (0.8)], hyperorality and appetite changes [74%, 1.1 (0.9)] and disinhibition [74%, 1.0 (0.8)], but all symptoms were significantly more severe than controls (Fig. 2A–E, Supplementary Table 3). In sporadic nfvPPA, the most frequent and severe symptoms were hyperorality and appetite changes [69%, 0.9 (0.9)], apathy [69%, 0.6 (0.5)] and poor response to emotional cues [56%, 0.9 (0.9)], with all symptoms except disinhibition and inappropriate trusting behaviour significantly more severe than controls. The lvPPA group had the least frequent and severe symptoms, with only apathy significantly more severe than controls [50%, 0.7 (0.8)].

In contrast to the sporadic PPA groups, the most common and severe behavioural symptom in both *GRN*-NFV and *GRN*-NOS groups was apathy [75%, 0.9 (0.6); 67%, 0.8 (0.8), respectively]. Both groups also had significantly more severe loss of sympathy/empathy [50%, 0.4 (0.4); 56%, 0.4 (0.5), respectively] and hyperorality and appetite changes [75%, 0.8 (0.8); 44%, 0.7 (0.9), respectively] compared with controls. Additionally, in the *GRN*-NOS group, poor response to emotional cues was more severely affected than controls [56%, 0.6, (0.7)].

Neuropsychiatric symptoms occurred commonly in patients in each group compared with only 16% of controls: 100% of patients in all groups had symptoms except genetic nfvPPA (88%).

**Figure 3:**
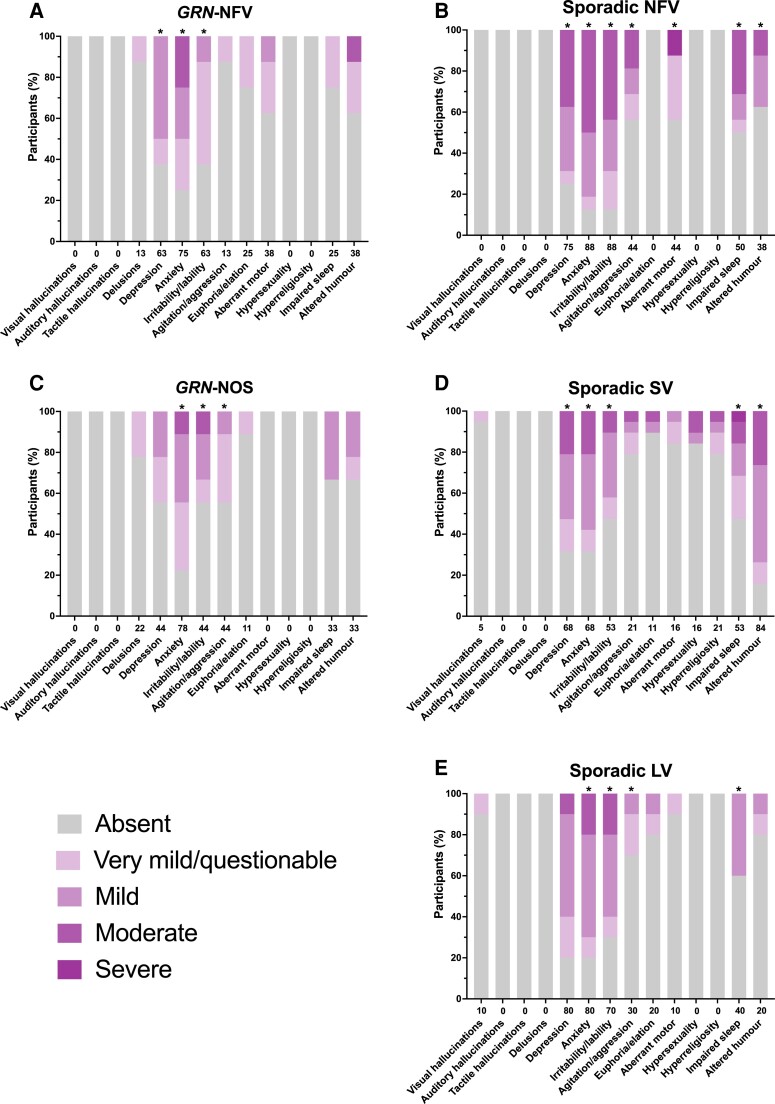
**Neuropsychiatric symptoms showing the percentage of participants in each of the PPA groups who score 0 = absent, 0.5 = very mild/questionable, 1 = mild, 2 = moderate, or 3 = severe for each symptom. A**, *GRN*-NFV; **B**, Sporadic NFV; **C**, *GRN*-NOS; **D**, Sporadic SV; **E**, Sporadic LV. Values along the x-axis represent the frequency (%) with which the symptom is present in any severity category (0.5–3). An asterisk above the bar indicates that the symptom severity is significantly greater than controls. Linear regressions adjusting for age and sex were used to compare groups for each symptom: visual hallucinations [Wald chi^2^(7) = 3.3, *P* = 0.856; *R*^2^ = 0.117]; delusions [Wald chi^2^(7) = 4.6, *P* = 0.708; *R*^2^ = 0.084]; depression [Wald chi^2^(7) = 74.8, *P* < 0.001; *R*^2^ = 0.417]; anxiety [Wald chi^2^(7) = 118.4, *P* < 0.001; *R*^2^ = 0.479]; irritability/lability [Wald chi^2^(7) = 77.4, *P* < 0.001; *R*^2^ = 0.454]; agitation/aggression [Wald chi^2^(7) = 21.2, *P* = 0.004; *R*^2^ = 0.232]; euphoria/elation [Wald chi^2^(7) = 7.8, *P* = 0.354; *R*^2^ = 0.080]; aberrant motor behaviour [Wald chi^2^(7) = 16.1, *P* = 0.025; *R*^2^ = 0.248]; hypersexuality [Wald chi^2^(7) = 4.0, *P* = 781; *R*^2^ = 0.140]; hyperreligiosity [Wald chi^2^(7) = 4.3, *P* = 0.749; *R*^2^ = 0.157]; impaired sleep [Wald chi^2^(7) = 28.5, *P* < 0.001; *R*^2^ = 0.244]; altered sense of humour [Wald chi^2^(7) = 60.3, *P* < 0.001; *R*^2^ = 0.434]. Abbreviations: *GRN*-NFV, nonfluent variant PPA due to progranulin mutation; *GRN*-NOS, not otherwise specified PPA due to progranulin mutation; LV, logopenic variant PPA; NFV, nonfluent variant PPA; SV, semantic variant PPA.

For all groups, depression, anxiety and irritability/lability were the most frequent and severe symptoms (Fig. 3A–E, Supplementary Table 3). Depression occurred most frequently in the lvPPA group (80%) followed by sporadic nfvPPA (75%) and svPPA (68%), with the two genetic groups showing the lowest frequency and severity (*GRN*-NFV 63%, *GRN*-NOS 44%). Depression severity was more significant than controls in all groups except *GRN*-NOS. Anxiety and irritability/lability were also common (and significantly more severe than controls) in all groups: 88% and 88%, respectively, in sporadic nfvPPA, 80% and 70% in lvPPA, 78% and 44% in *GRN*-NOS, 75% and 63% in *GRN*-NFV and 68% and 53% in svPPA. Apart from agitation/aggression in sporadic nfvPPA and *GRN*-NOS, and aberrant motor behaviour in sporadic nfvPPA, all other symptoms occurred at a low frequency and were not significantly different to controls. Of note, however, delusions occurred in 22% of *GRN*-NOS, and 13% of *GRN*-NFV but did not occur in the sporadic PPA groups.

Motor symptoms occurred in each of the PPA groups (and in 6% of controls), most commonly in the sporadic nfvPPA group (100%) and less frequently in the other groups: lvPPA (70%), genetic PPA-NOS (56%), genetic nfvPPA (50%) and svPPA (32%).

**Figure 4:**
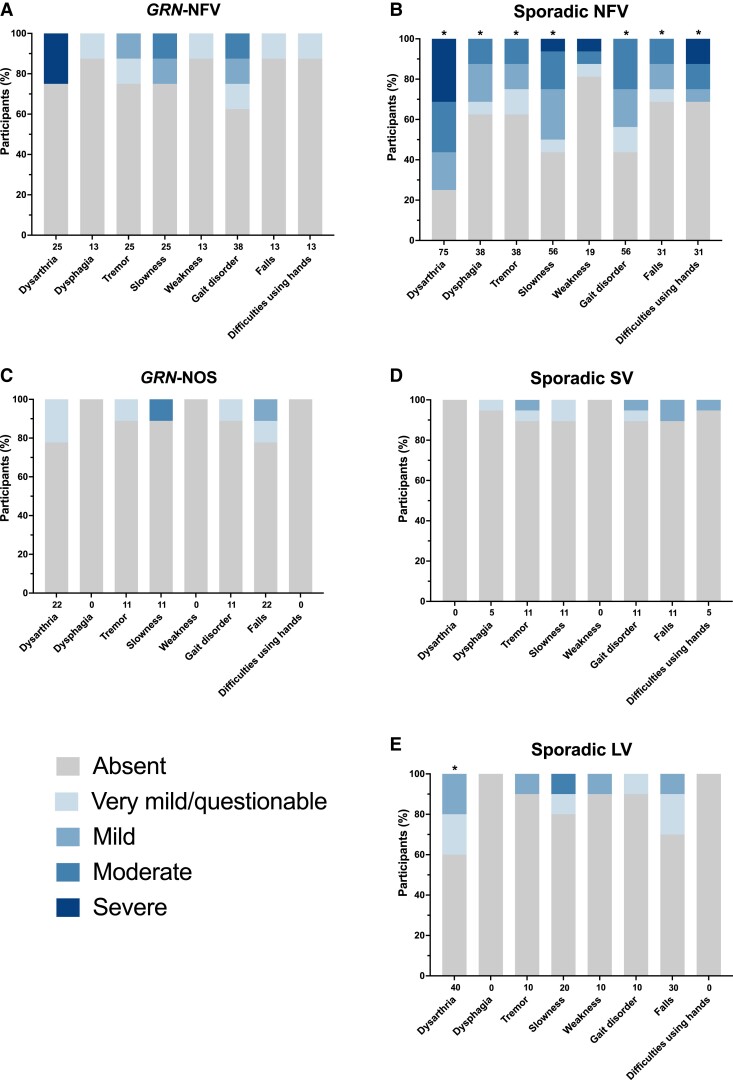
**Motor symptoms showing the percentage of participants in each of the PPA groups who score 0 = absent, 0.5 = very mild/questionable, 1 = mild, 2 = moderate, or 3 = severe for each symptom. A**, *GRN*-NFV; **B**, Sporadic NFV; **C**, *GRN*-NOS; **D**, Sporadic SV; **E**, Sporadic LV. Values along the x-axis represent the frequency (%) with which the symptom is present in any severity category (0.5–3). An asterisk above the bar indicates that the symptom severity is significantly greater than controls. Linear regressions adjusting for age and sex were used to compare groups for each symptom: dysarthria [Wald chi^2^(7) = 38.9, *P* < 0.001; *R*^2^ = 0.495]; dysphagia [Wald chi^2^(7) = 9.1, *P* = 0.244; *R*^2^ = 0.263]; tremor [Wald chi^2^(7) = 8.2, *P* = 0.312; *R*^2^ = 0.146]; slowness [Wald chi^2^(7) = 23.3, *P* = 0.002; *R*^2^ = 0.297]; weakness [Wald chi^2^(7) = 4.4, *P* = 0.734; *R*^2^ = 0.130]; gait disorder [Wald chi^2^(7) = 25.1, *P* = 0.001; *R*^2^ = 0.350]; falls [Wald chi^2^(7) = 14.8, *P* = 0.038; *R*^2^ = 0.180]; functional difficulties using hands [Wald chi^2^(7) = 8.8, *P* = 0.270; *R*^2^ = 0.246]. Abbreviations: *GRN*-NFV, nonfluent variant PPA due to progranulin mutation; *GRN*-NOS, not otherwise specified PPA due to progranulin mutation; LV, logopenic variant PPA; NFV, nonfluent variant PPA; SV, semantic variant PPA.

All motor symptoms apart from weakness occurred frequently and more severely than controls in the sporadic nfvPPA group with the most frequent and severe being dysarthria [75%, 1.6 (1.2)], slowness [56%, 0.8 (1.0)] and gait disorder [56%, 0.8 (0.8)] (Fig. 4A–E, Supplementary Table 3). Apart from dysarthria in the lvPPA group, no other symptom in any of the groups was significantly more severe than controls. In particular, although dysarthria (25%), gait disorder (38%) and slowness (25%) all occurred in the *GRN*-NFV group, this was less frequent than the sporadic nfvPPA group. Furthermore, 50% of the sporadic nfvPPA group symptoms met consensus criteria for one of the atypical parkinsonian disorders (four with PSP, three with CBS and one with a PSP/CBS overlap syndrome), whilst none of the other patients in any of the other groups met criteria.

#### Cognitive assessment

The mean score for the BNT was significantly different to controls for all PPA groups apart from the sporadic nfvPPA cohort where there was a trend for a lower score (*P* = 0.055) (Table 2). The sporadic svPPA group scored the lowest at 8.2 (8.1) and was significantly different to *GRN*-NFV, *GRN*-NOS and sporadic nfvPPA groups (*P* = 0.005, *P* = <0.001 and *P* = <0.001, respectively). The sporadic lvPPA group also had a significantly lower score of 12.7 (6.8) compared to *GRN*-NOS and sporadic nfvPPA groups (*P* = 0.014, *P* = <0.001, respectively). Scores in the two genetic PPA groups were lower than controls but lay between the sporadic nfvPPA group (23.6 (5.8)) and the other two sporadic PPA groups: *GRN*-NFV 18.0 (8.5), *GRN*-NOS 21.4 (7.0).

**Table 2:** Neuropsychological assessment scores in the genetic and sporadic PPA subgroups as well as healthy controls

	Controls	Genetic PPA		Sporadic PPA		
		*GRN*-NFV	*GRN*-NOS	LV	NFV	SV
Boston Naming Test (/30)	27.3 (2.1)	**18.0^a^ (8.5)**	**21.4** (**7.0)^b^**	**12.7 (6.8)^c,d^**	23.6 (5.8)	**8.2 (8.1)^c,d,e^**
Modified Camel and Cactus Test (/32)	29.6 (1.7)	26.6 (6.6)	**22.0 (8.1)^c,f^**	29.5 (0.7)	28.5 (4.5)	25.2 (4.6)
Trail Making Test Part A (max 150 s)	36.6 (17.5)	**73.8** (**41.3)**	**69.1** (**40.5)^b^**	**101.0 (72.3)^b^**	60.3 (37.0)	**41.9 (20.0)**
Trail Making Test Part B (max 300 s)	88.4 (45.8)	**230.0** (**85.4)^b^**	**244.0 (93.8)^b,c^**	**225.2 (83.0)^b^**	**145.1** (**83.2)**	121.6 (99.5)
D-KEFS Color-Word Inference Test (max 180 s)	53.6 (13.2)	**132.8** (**49.9)^b^**	**127.4** (**55.7)^b^**	**145.7 (42.6)^b^**	**116.2** (**37.6)^b^**	**78.7 (34.8)**
Digit Symbol test (max in 90 s)	49.2 (12.1)	**25.3** (**15.9)^b^**	**28.1** (**15.2)^b^**	**20.6 (10.3)^b^**	**33.8** (**13.4)**	**41.8 (15.6)**
Digit Span Forwards (/12)	7.3 (1.8)	**3.1** (**1.5)^b^**	**5.0** (**2.8)^b^**	**3.2 (2.3)^b^**	**5.1** (**2.2)^b^**	8.1 (3.0)
Digit Span Backwards (/12)	6.4 (1.9)	**2.7** (**1.7)^b^**	**3.1** (**2.6)^b^**	**2.2 (1.2)^b^**	**3.5** (**1.6)^b^**	6.5 (3.2)
Category fluency (max in 60 s)	22.7 (6.1)	**13.9** (**5.4)**	**11.8** (**6.5)**	**4.4 (3.3)^c,d,e^**	**10.1** (**6.4)**	**6.8 (5.1)^e^**
Block Design (/71)	41.7 (11.4)	**20.3** (**17.4)^b^**	**22.3** (**16.2)^b^**	**13.2 (10.7)^b,c^**	**26.3** (**20.3)**	37.8 (18.8)
FCSRT—free recall (/48)	27.3 (7.9)	33.5 (7.8)	**19.6** (**5.9)^e^**			
FCSRT—free + cued recall (/48)	44.3 (5.9)	46.5 (2.1)	**40.2** (**4.1)^e^**			
FCSRT—free delayed recall (/16)	10.8 (3.1)	12.5 (4.9)	8.4 (2.3)			
FCSRT—free + cued delayed recall (/16)	14.9 (1.8)	16.0 (0.0)	13.6 (2.1)^e^			
Mini-SEA: Faux Pas test (/40)	34.3 (5.2)	**25.0** (**7.1)**	**25.5** (**8.9)**			
Mini-SEA: Facial Emotion Recognition Test (/35)	27.9 (3.4)	**21.3** (**5.3)**	**22.1** (**7.5)**			

All data are shown as mean (standard deviation).

*GRN*-NFV, nonfluent variant PPA due to progranulin mutation; *GRN*-NOS, not otherwise specified PPA due to progranulin mutation; LV, logopenic variant PPA; NFV, nonfluent variant PPA; SV, semantic variant PPA. FCSRT, Free and Cued Selective Reminding Test; Mini-SEA; Mini-Social Cognition and Emotion Assessment.

a Bold items are significantly different to controls.

b Significantly impaired compared to svPPA.

c Significantly impaired compared to nfvPPA.

d Significantly impaired compared to *GRN*-NOS.

e Significantly impaired compared to *GRN*-NFV.

f Significantly impaired compared to lvPPA.

The mean score for the mCCT was significantly lower in the *GRN*-NOS group compared with controls [22.0 (8.1), *P* = 0.001] with a trend in the svPPA group [25.2 (4.6), *P* = 0.073]. The *GRN*-NOS group scored significantly lower than the lvPPA [29.5 (0.7), *P* = 0.007] and sporadic nfvPPA groups [28.5 (4.5), *P* = 0.025].

All groups performed significantly worse than controls on the category fluency task, with the lowest scores being found in the lvPPA [4.4 (3.3)] and svPPA [6.8 (5.1)] groups.

In the other cognitive tests, executive dysfunction was present in all groups, although to a lesser extent in the svPPA group, with significantly worse performance than controls in the D-KEFS CWIT and Digit Symbol test for all groups and for the TMTB and DSB in all groups except svPPA. Similarly, all groups except the svPPA cohort showed significant impairment compared to controls on DSF and the Block Design task.

In the tests performed only in the genetic PPA cohort, there was evidence of impaired episodic memory in the *GRN*-NOS group, with significantly lower scores compared to controls for both FCSRT free recall and free and cued recall (*P* = 0.034 and *P* = 0.009, respectively), and significantly lower scores than *GRN*-NFV individuals for all FCSRT tests except free delayed recall (*P* = 0.073). Both the *GRN*-NFV and *GRN*-NOS groups scored significantly lower on both parts of the mini-SEA: 25.0 (7.1) and 25.5 (8.9) for the Faux Pas test and 21.3 (5.3) and 22.1 (7.5) for the Facial Emotion Recognition Test.

#### Imaging analysis

**Figure 5:**
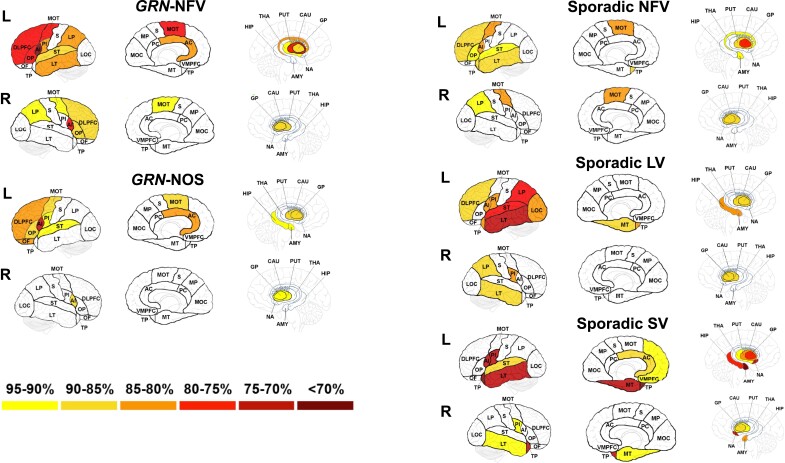
**Percentage regional brain volumes in each PPA group compared with controls.** These were calculated by dividing the mean of each regional brain volume in each PPA group by the mean of the same regional brain volume in the control group and multiplying by 100. The darkest colours represent areas of lowest brain volume as per the key. Abbreviations: *GRN*-NFV, nonfluent variant PPA due to progranulin mutation; *GRN*-NOS, not otherwise specified PPA due to progranulin mutation; LV, logopenic variant PPA; NFV, nonfluent variant PPA; SV, semantic variant PPA; AMY, amygdala; AC, anterior cingulate; AI, anterior insula; CAU, caudate; DLPFC, dorsolateral prefrontal cortex; GP, globus pallidus; HIP, hippocampus; LOC, lateral occipital; LP, lateral parietal; LT, lateral temporal; MOC, medial occipital; MP, medial parietal; MT, medial temporal; MOT, motor; NA, nucleus accumbens; OF, orbitofrontal; OP, opercular; PC, posterior cingulate; PI, posterior insula; PUT, putamen; S, somatosensory; ST, supratemporal; THA, thalamus; TP, temporal pole; VMPFC, ventromedial prefrontal cortex.

Patterns of brain atrophy in the sporadic PPA cohorts were as expected with the most atrophied regions being the left anterior insula (83% of mean control volume in that region), left motor (85%) and dorsolateral prefrontal cortices (87%), and the left basal ganglia (globus pallidus 79%, putamen 87%) in nfvPPA; left > right temporal pole (52%, 73%), left amygdala (58%), left hippocampus (77%) and left nucleus accumbens (75%) in svPPA; and left lateral temporal (74%), supratemporal (77%) and lateral parietal cortices (80%) in lvPPA (Fig. 5, Supplementary Table 4).

The pattern of atrophy was similar in the *GRN*-NFV group compared with the sporadic nfvPPA group with the most affected regions being the left anterior insula (67%), left motor (80%) and left dorsolateral prefrontal cortices (76%) as well as basal ganglia involvement (globus pallidus 86%, putamen 80%). More posterior involvement was also seen in the *GRN*-NFV group with left lateral parietal volume significantly lower than controls (83%).

The pattern of atrophy was different in the *GRN*-NOS group with a highly asymmetrical pattern affecting the left hemisphere particularly anterior insula (75%), dorsolateral prefrontal cortex (82%) and, unlike the other PPA groups, the orbitofrontal cortex (85%). Other areas that were significantly different to controls included the amygdala and hippocampus as well as the basal ganglia.

## Discussion

In this study, we have shown that, although there are overlapping symptoms between genetic and sporadic PPA syndromes, there are also distinct features. PPA is a common clinical syndrome in those with *GRN* mutations (44% in the GENFI cohort) but uncommon in both *C9orf72* and *MAPT* mutations (only one case in the GENFI cohort in both genetic groups). Within the *GRN* mutation carriers, there seem to be two distinct groups, a nfvPPA group which overlaps to a large extent with sporadic nfvPPA, although at least in this cohort with fewer atypical parkinsonian motor features, and a PPA-NOS group, not neatly fitting into any of the canonical sporadic syndromes, with highly asymmetrical left hemisphere atrophy on brain imaging.

The linguistic symptoms in our comparator sporadic PPA cohort were consistent with previously described patterns of impairment.^2,22^ Prior studies of PPA in *GRN* mutations have been relatively small, but here, we showed that two groups emerge, one with nfvPPA and one with a PPA-NOS syndrome. The pattern of deficits in the genetic nfvPPA group was similar to that of sporadic nfvPPA although with quite severe dysgraphia also in the genetic group. The PPA-NOS group was distinct—features seen in each of the canonical PPA were present: impaired grammar/syntax similar to nfvPPA (but no deficit in articulation), impaired word retrieval and speech repetition similar to lvPPA and impaired single word comprehension and semantic deficits (seen on the mCCT) similar to svPPA. Sentence comprehension deficits (seen in both nfvPPA and lvPPA) were also prominent in the *GRN*-NOS group. In other words, the PPA-NOS group here have mixed features of the different PPA canonical syndromes but distinctively have impaired grammar/syntax in the presence of relatively intact articulation.

Our findings contrast with a previous study of *GRN*-associated PPA in which they found 28% of the cohort had a diagnosis of nfvPPA, 25% had a ‘mixed’ PPA and 41% held a diagnosis of lvPPA.^8^ One reason for this may be that people with lvPPA are often assumed to have AD pathologically and so less genetic testing is done in some centres. However, this study was a retrospective analysis, and half of their lvPPA patients were said to have an ‘lvPPA plus’ syndrome, i.e. features of another form of PPA including semantic impairment and syntax difficulties. In fact, their findings overlap with this study in that both the ‘mixed’ and ‘lvPPA plus’ groups have a similar pattern of deficits to those seen in the PPA-NOS group here.

Behavioural symptoms were present in the *GRN*-PPA groups with a similar frequency and severity to sporadic svPPA and nfvPPA but more frequent than lvPPA (which is usually an atypical AD syndrome). Apathy was the most common behavioural symptom in both *GRN*-NFV and *GRN*-NOS, consistent with prior reports of this being a frequent feature in people with *GRN* mutations.^31^

Like the other PPA groups, depression, anxiety and irritability/lability were common symptoms in the *GRN*-PPA groups, although depression was less frequent in the *GRN*-NOS group. Strikingly, although occurring at a low frequency, delusions were only seen in the two *GRN*-PPA groups and not the sporadic PPA groups. Although often felt to be characteristic of *C9orf72* mutations, delusions have previously been reported in a significant minority of people with *GRN* mutations.^32^

As with previous reports, this study found that sporadic nfvPPA was associated with the most frequent and severe motor symptoms, with 50% having features consistent with an atypical parkinsonian syndrome that developed between 1 and 5 years after initial language symptoms, either PSP or CBS (or in one case an overlap of both).^33–36^ Similar motor features were seen in the *GRN*-NFV group but to a lesser extent and without anyone in this study meeting criteria for an atypical parkinsonian syndrome. Extrapyramidal features have been previously described in *GRN* mutations (in up to 40–60% of patients^37,38^), and people with overlapping syndromes of PPA and CBS have also been seen, but PSP is not a syndrome seen in *GRN* mutations.^39^ The less frequent occurrence of motor features and full-blown parkinsonian syndromes (particularly PSP) appears to be a distinct feature of the *GRN* nfvPPA form.

Non-linguistic cognitive deficits in the *GRN*-PPA groups were similar to the other groups, particularly the nfvPPA cohort and to a lesser extent the lvPPA cohort, with significant executive dysfunction and impaired category fluency. It may be that this deficit also contributed to the poor performance of the *GRN*-NOS group in the mCCT, which is known to have an executive component.^40,41^ However, there was also impairment in Digit Span Forwards in both *GRN*-PPA groups, particularly in the *GRN*-NOS group, potentially indicative of more lateral parietal cortical deficits in these groups.^42^

Interestingly, the *GRN*-NOS group had lower scores for the FCSRT suggesting more episodic memory impairment in this group compared with *GRN*-NFV individuals and consistent with the finding of hippocampal atrophy in this cohort. Although not tested here, impairment of episodic memory is more common in lvPPA and not generally seen in nfvPPA and svPPA.^43,44^

Both *GRN*-PPA groups scored significantly worse than controls on the tests of social cognition, the Faux Pas test (testing theory of mind) and Facial Emotion Recognition Test. This is consistent with prior studies showing impaired social cognition in PPA.^45–47^

Patterns of atrophy in the sporadic PPA subtypes were as previously described.^48,49^ In previous studies of *GRN*-associated PPA, a highly asymmetrical left-sided predominant atrophy pattern has been described,^1,50^ with the insula being the first brain region to be affected, up to 15 years prior to onset of symptomatic disease.^51^ However, in our study, we were able to separate out two groups (*GRN*-NFV and *GRN*-NOS). The pattern in the *GRN*-NFV group overlapped with that of the sporadic nfvPPA cohort, although with more posterior cortical involvement of the lateral parietal cortex. In contrast, the *GRN*-NOS group was strikingly asymmetrical with involvement of the insula and dorsolateral prefrontal cortex (similar to the *GRN*-NFV group) but also atrophy of the orbitofrontal cortex (in distinction to the other forms of PPA) and the medial temporal lobes (amygdala and hippocampus).

In summary, we have described a genetic nfvPPA group similar to sporadic nfvPPA with decreased fluency, impaired grammar/syntax and impaired articulation, but with relatively intact single word comprehension, along with an overlapping pattern of atrophy. The only distinctions in these syndromes were in the non-linguistic features, with delusions occurring only in the *GRN* group and more prominent motor features and atypical parkinsonian syndromes in the sporadic nfvPPA group. We have also described a *GRN*-NOS group, similar to what has been described previously as a mixed form of PPA, with linguistic features that are seen in each of the sporadic PPA syndromes but distinctively grammar/syntax problems alongside relatively intact articulation. As with *GRN*-NFV, delusions are seen in a minority of individuals, but other behavioural and neuropsychiatric features are not particularly specific (although are more prominent than in lvPPA which this may be mistaken for clinically, particularly as there can be episodic memory impairment). The pattern of atrophy is also overlapping but with unique features including involvement of the orbitofrontal cortex as well as the medial temporal lobe.

We only found one patient with a *C9orf72* mutation and one patient with a *MAPT* mutation who had a PPA syndrome (Supplementary Table 1). *C9orf72*-associated PPA is very uncommon^39^ with a recent review finding only a small number of cases reported in the literature.^9^ The clinical syndrome is usually nfvPPA but can also be svPPA or, like here, a PPA-NOS. Similarly, *MAPT*-associated PPA is also very uncommon. Whilst many people with a bvFTD syndrome have associated semantic deficits,^52^ it is rare for such an impairment to be the primary symptom, leading to a diagnosis of svPPA as seen here.

### Limitations

Firstly, despite the large numbers overall compared with previous studies, when stratified, the numbers in the individual groups are small. Nonetheless, this work provides important evidence about the phenotype of *GRN*-related PPA which will need to be substantiated in future prospective cohort studies. Secondly, linguistic symptom data was limited to the 10 items of the PASS, and whilst this encompasses the majority of symptoms relevant to the canonical PPA syndromes, it does not separate out single word and sentence repetition (with just a single ‘speech repetition’ item), which can be helpful in differentiating nfvPPA and lvPPA. Furthermore, we only had data from two linguistic neuropsychometric tests (BNT and mCCT). This is because the GENFI neuropsychology battery was designed to include mainly visual tasks due to the involvement of countries speaking eight different languages and the difficulty of translating and validating verbal tasks across those languages. In future studies it would be helpful to consider developing a multilanguage verbal linguistic battery to study cognitive deficits further in this cohort. Thirdly, we only had data on episodic memory and social cognition in the genetic groups which limited the comparison with the canonical groups. Lastly, we focused on cross-sectional differences, but longitudinal analysis would be helpful in future studies to understand the progression of disease with additional symptoms and also the presence of very early deficits, particularly in the prodromal genetic cohort.

In conclusion, we examined the presence of PPA syndromes in the GENFI cohort and found that the language network was strikingly more vulnerable to *GRN* mutations than either *C9orf72* or *MAPT* mutations. Two distinct PPA syndromes were found in association with *GRN* mutations - those with nfvPPA, similar to sporadic nfvPPA, and those with a PPA-NOS, who had mixed features overlapping with the canonical sporadic disorders but, distinctively, impaired grammar/syntax in the presence of relatively intact articulation. Better recognition of the phenotypic presentations of *GRN*-related PPA are likely to trigger earlier genetic testing and in turn lead to improvements in management, including allowing people to enter clinical trials for those with *GRN* mutations and family members to access genetic counselling. We recommend that future iterations of the consensus criteria for PPA encompass such information, with the suggestion of testing for a *GRN* mutation, particularly in those with such a phenotype. Future work will need to understand the earliest stages of these disorders and how they initially present in the prodromal period within the GENFI cohort, allowing us to better stage these disorders and identify people at a time when they can potentially most benefit within clinical trials.

## Supplementary material

Supplementary material is available at *Brain Communications* online.

## Supplementary Material

fcad036_Supplementary_Data

## Appendix I

### GENFI Consortium (please see Supplementary Appendix for Consortia affiliations)

- Sónia Afonso
- Maria Rosario Almeida
- Sarah Anderl-Straub
- Christin Andersson
- Anna Antonell
- Silvana Archetti
- Andrea Arighi
- Mircea Balasa
- Myriam Barandiaran
- Nuria Bargalló
- Robart Bartha
- Benjamin Bender
- Alberto Benussi
- Maxime Bertoux
- Valentina Bessi
- Sandra Black
- Sergi Borrego-Ecija
- Jose Bras
- Alexis Brice
- Rose Bruffaerts
- Agnès Camuzat
- Marta Cañada
- Valentina Cantoni
- Paola Caroppo
- Miguel Castelo-Branco
- Olivier Colliot
- Thomas Cope
- Vincent Deramecourt
- María de Arriba
- Giuseppe Di Fede
- Alina Díez
- Diana Duro
- Chiara Fenoglio
- Camilla Ferrari
- Catarina B. Ferreira
- Nick Fox
- Morris Freedman
- Giorgio Fumagalli
- Aurélie Funkiewiez
- Alazne Gabilondo
- Roberto Gasparotti
- Serge Gauthier
- Stefano Gazzina
- Giorgio Giaccone
- Ana Gorostidi
- Rita Guerreiro
- Carolin Heller
- Tobias Hoegen
- Begoña Indakoetxea
- Vesna Jelic
- Hans-Otto Karnath
- Ron Keren
- Tobias Langheinrich
- Maria João Leitão
- Albert Lladó
- Gemma Lombardi
- Sandra Loosli
- Carolina Maruta
- Simon Mead
- Lieke Meeter
- Gabriel Miltenberger
- Rick van Minkelen
- Sara Mitchell
- Katrina Moore
- Benedetta Nacmias
- Annabel Nelson
- Linn Öijerstedt
- Jaume Olives
- Sebastien Ourselin
- Alessandro Padovani
- Jessica Panman
- Janne M. Papma
- Yolande Pijnenburg
- Cristina Polito
- Enrico Premi
- Sara Prioni
- Catharina Prix
- Rosa Rademakers
- Veronica Redaelli
- Daisy Rinaldi
- Tim Rittman
- Adeline Rollin
- Pedro Rosa-Neto
- Giacomina Rossi
- Martin Rossor
- Beatriz Santiago
- Dario Saracino
- Sabrina Sayah
- Elio Scarpini
- Sonja Schönecker
- Elisa Semler
- Rachelle Shafei
- Christen Shoesmith
- Imogen Swift
- Miguel Tábuas-Pereira
- Mikel Tainta
- Ricardo Taipa
- David Tang-Wai
- David L. Thomas
- Paul Thompson
- Hakan Thonberg
- Carolyn Timberlake
- Pietro Tiraboschi
- Emily Todd
- Philip Van Damme
- Mathieu Vandenbulcke
- Michele Veldsman
- Ana Verdelho
- Jorge Villanua
- Carlo Wilke
- Ione Woollacott
- Elisabeth Wlasich
- Henrik Zetterberg
- Miren Zulaica

## Abbreviations

AD =: Alzheimer’s disease
AMY =: amygdala
AC =: anterior cingulate
AI =: anterior insula
bvFTD =: behavioural variant frontotemporal dementia
BNT =: Boston Naming Test
CAU =: caudate
*C9orf72* =: chromosome 9 open reading frame 72
CDR =: Clinical Dementia Rating
CBS =: corticobasal syndrome
CWIT =: Color-Word Interference Test
DLPFC =: dorsolateral prefrontal cortex
FCSRT =: Free and Cued Selective Reminding Test
FTD =: frontotemporal dementia
GENFI =: GENetic Frontotemporal dementia Initiative
GIF =: geodesic information flow
GP =: globus pallidus
HIP =: hippocampus
LOC =: lateral occipital
LP =: lateral parietal
LT =: lateral temporal
lvPPA =: logopenic variant primary progressive aphasia
LIFTD =: longitudinal investigation of frontotemporal dementia
MOC =: medial occipital
MP =: medial parietal
MT =: medial temporal
*MAPT* =: microtubule-associated protein tau
MMSE =: Mini-Mental State Examination
mini-SEA =: Mini-Social cognition and Emotion Assessment
mCCT =: Modified Camel and Cactus Test
MT =: motor
nfvPPA =: nonfluent variant primary progressive aphasia
NA =: nucleus accumbens
OF =: orbitofrontal
OP =: opercular
PC =: posterior cingulate
PI =: posterior insula
PPA =: primary progressive aphasia
PPA -NOS =: Primary progressive aphasia-not otherwise specified
*GRN* =: progranulin
*GRN*-NFV =: progranulin nonfluent variant
*GRN*-NOS =: progranulin-not otherwise specified
PASS =: Progressive Aphasia Severity Scale
PSP =: progressive supranuclear palsy
PUT =: putamen
svPPA =: semantic variant primary progressive aphasia
S =: somatosensory
ST =: supratemporal
THA =: thalamus
TP =: temporal pole
TIV =: total intracranial volume
TMT =: Trail Making Test
VMPFC =: ventromedial prefrontal cortex
DSF/DSB =: WMS-R Digit Span Forward/Backward
