# Supplementary material for: Genetic forms of primary progressive aphasia within the GENetic Frontotemporal dementia Initiative (GENFI) cohort: comparison with sporadic primary progressive aphasia
Source: Brain Commun. 2023 Feb 17;5(2):fcad036. doi: 10.1093/braincomms/fcad036 (PMC10019761; doi:10.1093/braincomms/fcad036)
Supplement: fcad036_Supplementary_Data [file fcad036_supplementary_data.pdf]

Supplementary Material

Samra et al. Genetic forms of primary progressive aphasia within the GENetic Frontotemporal dementia Initiative cohort – comparison with sporadic primary progressive aphasia.

Supplementary Table 1 Clinical features of the *C9orf72*-PPA and *MAPT*-PPA cases

	C9orf72	***MAPT***
**Mutation**	N/A	*P397S*
**Clinical syndrome**	PPA-NOS	svPPA
**Age at onset**	50s	50s
**Progressive aphasia severity scale sum of boxes**	6	3.5
**Impaired articulation**	Absent	Absent
**Decreased fluency**	Mild	Absent
**Impaired grammar/syntax**	Mild	Absent
**Impaired word retrieval**	Mild	Moderate
**Impaired speech repetition**	Moderate	Absent
**Impaired sentence comprehension**	Mild	Absent
**Impaired single word comprehension**	Absent	Mild
**Dyslexia**	Absent	Absent
**Dysgraphia**	Absent	Absent
**Impaired function communication**	Mild	Very mild/questionable
**BNT (/30)**	11	16
**mCCT (/32)**	15	25
**Other symptoms**	Apathy	Disinhibition<br>Abnormal trusting behaviour<br>Impaired episodic memory<br>Apathy

Supplementary Table 2 Results summary of the ordinal logistic regression comparing linguistic symptoms across the PPA groups. P-values are shown in parentheses. Abbreviations: *GRN*-NFV, nonfluent variant PPA due to progranulin mutation; *GRN*-NOS, not otherwise specified PPA due to progranulin mutation; LV, logopenic variant PPA; NFV, nonfluent variant PPA; SV, semantic variant PPA. Higher score in each symptom means more impaired.

	**Genetic PPA**		**Sporadic PPA**		
	***GRN*-NFV**	***GRN*-NOS**	**LV**	**NFV**	**SV**
**Impaired articulation**	Higher than *GRN*-NOS (0.001), LV (<0.001), SV (<0.001)	N/A	Higher than SV (0.002)	Higher than *GRN*-NOS, LV, and SV (<0.001)	N/A
**Decreased fluency**	Higher than LV and SV (<0.001)	Higher than SV (<0.001)	Higher than SV (0.038)	Higher than LV and SV (<0.001)	N/A
**Impaired grammar/syntax**	Higher than LV and SV (<0.001)	Higher than LV (0.012) and SV (<0.001)	N/A	Higher than LV and SV (<0.001)	N/A
**Impaired word retrieval**	N/A	N/A	N/A	N/A	N/A
**Impaired speech repetition**	Higher than SV (0.003)	Higher than SV (0.014)	Higher than SV (<0.001)	Higher than SV (<0.001)	N/A
**Impaired sentence comprehension**	N/A	Higher than *GRN*-NFV (0.026)	N/A	N/A	Higher than *GRN*-NFV (0.036)
**Impaired single word comprehension**	N/A	N/A	Higher than *GRN*-NFV (0.036)	N/A	Higher than *GRN*-NFV, LV, and NFV (<0.001), and *GRN*-NOS (0.005)
**Dyslexia**	N/A	N/A	N/A	N/A	Higher than *GRN*-NOS (0.012)
**Dysgraphia**	Higher than *GRN*-NOS and LV (0.002), and SV (0.009)	N/A	N/A	Higher than *GRN*-NOS (0.031) and LV (0.045)	N/A
**Impaired function communication**	Higher than SV (<0.001)	N/A	N/A	Higher than *GRN*-NOS (0.006), LV (0.001), and SV (<0.001)	N/A

Supplementary Table 3 Mean (standard deviation) severity scores for the non-linguistic symptoms. Abbreviations: *GRN*-NFV, nonfluent variant PPA due to progranulin mutation; *GRN*-NOS, not otherwise specified PPA due to progranulin mutation; LV, logopenic variant PPA; NFV, nonfluent variant PPA; SV, semantic variant PPA. Bold items are significantly different to controls.

	Controls	Genetic PPA		Sporadic PPA		
		***GRN*-NFV**	***GRN*-NOS**	**LV**	**NFV**	**SV**
Behavioural symptoms						
**Disinhibition**	0.0 (0.1)	0.2 (0.3)	0.2 (0.4)	0.1 (0.2)	0.2 (0.4)	**1.0 (0.8)**
**Apathy**	0.0 (0.0)	**0.9 (0.6)**	**0.8 (0.8)**	**0.7 (0.8)**	**0.6 (0.5)**	**0.5 (0.7)**
**Loss of sympathy/empathy**	0.0 (0.0)	**0.4 (0.4)**	**0.4 (0.5)**	0.4 (0.7)	**0.8 (0.8)**	**1.1 (0.8)**
**Ritualistic/compulsive behaviour**	0.0 (0.0)	0.2 (0.3)	0.2 (0.7)	0.0 (0.0)	**0.7 (0.8)**	**0.9 (0.9)**
**Hyperorality and appetite changes**	0.0 (0.2)	**0.8 (0.8)**	**0.7 (0.9)**	0.4 (0.5)	**0.9 (0.9)**	**1.1 (0.9)**
**Poor response to emotional cues**	0.0 (0.0)	0.2 (0.4)	**0.6 (0.7)**	0.3 (0.7)	**0.9 (0.9)**	**0.8 (0.9)**
**Inappropriate trusting behaviour**	0.0 (0.1)	0.3 (0.4)	0.2 (0.7)	0.0 (0.6)	0.1 (0.7)	**0.9 (0.7)**
Neuropsychiatric symptoms						
**Visual hallucinations**	0.0 (0.0)	0.0 (0.0)	0.0 (0.0)	0.1 (0.2)	0.0 (0.0)	0.0 (0.1)
**Auditory hallucinations**	0.0 (0.0)	0.0 (0.0)	0.0 (0.0)	0.0 (0.0)	0.0 (0.0)	0.0 (0.0)
**Tactile hallucinations**	0.0 (0.0)	0.0 (0.0)	0.0 (0.0)	0.0 (0.0)	0.0 (0.0)	0.0 (0.0)
**Delusions**	0.0 (0.1)	0.1 (0.2)	0.1 (0.2)	0.0 (0.0)	0.0 (0.0)	0.0 (0.0)
**Depression**	0.0 (0.2)	**0.6 (0.5)**	0.3 (0.4)	**0.8 (0.6)**	**1.1 (0.8)**	**0.8 (0.8)**
**Anxiety**	0.0 (0.1)	**0.9 (0.8)**	**0.7 (0.6)**	**1.0 (0.7)**	**1.3 (0.7)**	**0.8 (0.7)**
**Irritability/lability**	0.0 (0.1)	**0.4 (0.4)**	**0.5 (0.7)**	**0.9 (0.7)**	**1.2 (0.8)**	**0.6 (0.7)**
**Agitation/aggression**	0.0 (0.0)	0.1 (0.2)	**0.3 (0.4)**	0.2 (0.4)	**0.6 (0.8)**	0.2 (0.5)
**Euphoria/elation**	0.0 (0.0)	0.1 (0.2)	0.1 (0.2)	0.2 (0.3)	0.0 (0.0)	0.2 (0.5)
**Aberrant motor behaviour**	0.0 (0.0)	0.3 (0.4)	0.0 (0.0)	0.1 (0.2)	**0.7 (1.0)**	0.2 (0.5)
**Hypersexuality**	0.0 (0.0)	0.0 (0.0)	0.0 (0.0)	0.0 (0.0)	0.0 (0.0)	0.3 (0.7)
**Hyperreligiosity**	0.0 (0.0)	0.0 (0.0)	0.0 (0.0)	0.0 (0.0)	0.0 (0.0)	0.2 (0.5)
**Impaired sleep**	0.1 (0.2)	0.3 (0.5)	0.3 (0.5)	**0.4 (0.5)**	**0.8 (0.9)**	**0.6 (0.9)**
**Altered sense of humour**	0.0 (0.0)	0.4 (0.7)	0.3 (0.4)	0.2 (0.3)	**0.5. (0.7)**	**1.1 (0.7)**

Motor symptoms						
**Dysarthria**	0.0 (0.0)	0.8 (1.4)	0.1 (0.2)	**0.3 (0.4)**	**1.6 (1.2)**	0.0 (0.0)
**Dysphagia**	0.0 (0.0)	0.1 (0.2)	0.0 (0.0)	0.0 (0.0)	**0.5 (0.7)**	0.0 (0.11)
**Tremor**	0.0 (0.2)	0.2 (0.4)	0.1 (0.3)	0.1 (0.3)	**0.4 (0.7)**	0.1 (0.2)
**Slowness**	0.0 (0.1)	0.4 (0.7)	0.2 (0.7)	0.3 (0.6)	**0.8 (1.0)**	0.1 (0.2)
**Weakness**	0.0 (0.0)	0.1 (0.2)	0.0 (0.0)	0.1 (0.3)	0.3 (0.9)	0.0 (0.0)
**Gait disorder**	0.0 (0.1)	0.4 (0.7)	0.1 (0.3)	0.1 (0.3)	**0.8 (0.8)**	0.1 (0.3)
**Falls**	0.0 (0.0)	0.1 (0.2)	0.2 (0.4)	0.2 (0.4)	**0.4 (0.7)**	0.1 (0.3)
**Functional difficulties using hands**	0.0 (0.0)	0.1 (0.2)	0.0 (0.0)	0.0 (0.0)	**0.7 (1.1)**	0.1 (0.2)

Supplementary Table 4 Volumes (as a percentage of total intracranial volume, in mm^3) of cortical and subcortical brain regions in the genetic and sporadic primary progressive aphasia (PPA) subgroups as well as healthy controls. All data are shown as mean (standard deviation). Abbreviations: *GRN*-NFV, nonfluent variant PPA due to progranulin mutation; *GRN*-NOS, not otherwise specified PPA due to progranulin mutation; LV, logopenic variant PPA; NFV, nonfluent variant PPA; SV, semantic variant PPA. Bold items are significantly different to controls.

		Controls	Genetic PPA		Sporadic PPA		
			GRN-NFV	*GRN*-NOS	LV	NFV	SV
**Orbitofrontal**	**Left**	0.71 (0.05)	0.63 (0.07)	**0.60 (0.10)**	0.67 (0.06)	0.68 (0.07)	0.67 (0.09)
	**Right**	0.71 (0.04)	0.73 (0.06)	0.72 (0.10)	0.71 (0.04)	0.71 (0.08)	0.74 (0.07)
**Dorsolateral Prefrontal Cortex**	**Left**	2.71 (0.17)	**2.07 (0.22)**	**2.21 (0.38)**	**2.38 (0.13)**	**2.37 (0.28)**	2.62 (0.36)
	**Right**	2.66 (0.20)	**2.32 (0.17)**	2.52 (0.28)	2.44 (0.12)	2.41 (0.30)	2.67 (0.26)
**Ventromedial Prefrontal Cortex**	**Left**	0.68 (0.05)	0.63 (0.07)	0.62 (0.11)	0.69 (0.04)	0.66 (0.07)	**0.63 (0.09)**
	**Right**	0.81 (0.06)	0.83 (0.11)	0.82 (0.10)	0.88 (0.06)	0.81 (0.09)	0.82 (0.08)
**Motor**	**Left**	1.37 (0.10)	**1.09 (0.11)**	**1.20 (0.17)**	1.26 (0.11)	**1.16 (0.10)**	1.33 (0.13)
	**Right**	1.37 (0.10)	**1.27 (0.10)**	1.31 (0.12)	1.32 (0.11)	**1.16 (0.14)**	1.38 (0.12)
**Opercular**	**Left**	0.37 (0.03)	**0.28 (0.03)**	**0.33 (0.05)**	0.35 (0.03)	**0.35 (0.04)**	0.37 (0.04)
	**Right**	0.37 (0.03)	**0.32 (0.05)**	0.37 (0.03)	0.35 (0.02)	0.35 (0.04)	0.38 (0.03)
**Medial Temporal**	**Left**	0.96 (0.06)	0.96 (0.09)	0.95 (0.08)	**0.86 (0.08)**	0.97 (0.09)	**0.71 (0.07)**
	**Right**	0.97 (0.06)	1.00 (0.08)	0.99 (0.07)	0.95 (0.06)	1.01 (0.08)	**0.91 (0.08)**
**Lateral Temporal**	**Left**	2.26 (0.14)	**1.92 (0.25)**	2.02 (0.37)	**1.68 (0.12)**	**2.04 (0.24)**	**1.58 (0.21)**
	**Right**	2.29 (0.12)	2.22 (0.26)	2.28 (0.10)	**1.97 (0.20)**	2.21 (0.23)	**2.11 (0.17)**
**Temporal Pole**	**Left**	0.50 (0.05)	0.45 (0.07)	0.45 (0.10)	**0.41 (0.09)**	**0.44 (0.06)**	**0.26 (0.05)**
	**Right**	0.48 (0.05)	0.47 (0.06)	0.51 (0.05)	0.47 (0.06)	0.47 (0.07)	**0.35 (0.06)**
**Supratemporal**	**Left**	0.39 (0.04)	**0.35 (0.09)**	**0.36 (0.05)**	**0.30 (0.05)**	**0.37 (0.06)**	**0.34 (0.04)**
	**Right**	0.35 (0.03)	0.34 (0.04)	0.36 (0.01)	0.33 (0.03)	0.34 (0.05)	0.35 (0.04)
**Medial Parietal**	**Left**	0.64 (0.06)	0.63 (0.09)	0.63 (0.12)	0.60 (0.06)	0.65 (0.08)	0.68 (0.07)
	**Right**	0.64 (0.07)	0.65 (0.06)	0.68 (0.08)	0.63 (0.07)	0.65 (0.10)	0.74 (0.08)
**Lateral Parietal**	**Left**	1.78 (0.12)	**1.48 (0.25)**	1.64 (0.21)	**1.43 (0.11)**	1.65 (0.18)	1.72 (0.21)
	**Right**	1.78 (0.12)	**1.65 (0.13)**	1.83 (0.11)	**1.58 (0.10)**	**1.66 (0.20)**	1.86 (0.13)
**Sensory**	**Left**	0.70 (0.07)	0.67 (0.10)	0.74 (0.06)	0.68 (0.04)	0.66 (0.11)	0.72 (0.07)
	**Right**	0.63 (0.07)	0.68 (0.08)	0.67 (0.06)	0.63 (0.07)	0.59 (0.08)	0.67 (0.06)
**Medial Occipital**	**Left**	1.29 (0.10)	1.33 (0.07)	1.35 (0.19)	1.22 (0.15)	1.27 (0.12)	1.32 (0.11)

	**Right**	1.33	(0.14)	1.45	(0.08)	1.42	(0.18)	1.39	(0.19)	1.35	(0.15)	1.46	(0.11)
**Lateral Occipital**	**Left**	1.14	(0.12)	1.09	(0.10)	1.10	(0.18)	**0.94**	**(0.16)**	1.01	(0.11)	1.09	(0.12)
	**Right**	1.10	(0.13)	1.09	(0.10)	1.12	(0.20)	0.98	(0.12)	0.99	(0.11)	1.14	(0.10)
**Anterior Cingulate**	**Left**	0.38	(0.04)	**0.31**	**(0.05)**	**0.32**	**(0.06)**	0.36	(0.04)	0.34	(0.04)	**0.33**	**(0.05)**
	**Right**	0.29	(0.04)	0.24	(0.05)	0.27	(0.05)	0.26	(0.03)	0.25	(0.04)	0.26	(0.05)
**Posterior Cingulate**	**Left**	0.32	(0.03)	0.32	(0.03)	0.29	(0.05)	0.33	(0.04)	0.32	(0.04)	0.34	(0.03)
	**Right**	0.31	(0.02)	0.33	(0.03)	0.31	(0.04)	0.34	(0.04)	0.32	(0.04)	0.36	(0.03)
**Anterior Insula**	**Left**	0.24	(0.03)	**0.16**	**(0.03)**	**0.18**	**(0.04)**	**0.20**	**(0.02)**	**0.20**	**(0.03)**	**0.17**	**(0.03)**
	**Right**	0.25	(0.02)	**0.20**	**(0.04)**	**0.22**	**(0.03)**	0.22	(0.02)	0.22	(0.03)	0.22	(0.03)
**Posterior Insula**	**Left**	0.11	(0.01)	**0.09**	**(0.01)**	**0.10**	**(0.02)**	**0.09**	**(0.01)**	0.10	(0.02)	**0.08**	**(0.01)**
	**Right**	0.12	(0.01)	0.11	(0.02)	0.12	(0.01)	**0.10**	**(0.01)**	0.11	(0.02)	**0.11**	**(0.01)**
**Amygdala**	**Left**	0.12	(0.01)	0.12	(0.01)	**0.11**	**(0.01)**	**0.10**	**(0.01)**	**0.11**	**(0.01)**	**0.07**	**(0.01)**
	**Right**	0.12	(0.01)	0.12	(0.01)	0.11	(0.00)	0.11	(0.01)	0.12	(0.01)	**0.10**	**(0.01)**
**Hippocampus**	**Left**	0.26	(0.02)	0.25	(0.03)	**0.24**	**(0.02)**	**0.22**	**(0.03)**	0.25	(0.03)	**0.20**	**(0.02)**
	**Right**	0.27	(0.02)	0.27	(0.04)	0.27	(0.02)	0.25	(0.02)	0.26	(0.03)	0.25	(0.03)
**Nucleus Accumbens**	**Left**	0.04	(0.00)	0.04	(0.00)	0.04	(0.00)	0.04	(0.00)	0.04	(0.00)	**0.03**	**(0.00)**
	**Right**	0.04	(0.00)	0.04	(0.00)	0.04	(0.00)	0.04	(0.00)	0.04	(0.00)	**0.03**	**(0.00)**
**Caudate**	**Left**	0.22	(0.03)	**0.18**	**(0.02)**	0.21	(0.03)	0.24	(0.04)	**0.21**	**(0.03)**	0.21	(0.03)
	**Right**	0.23	(0.03)	**0.22**	**(0.03)**	0.23	(0.04)	0.25	(0.03)	0.23	(0.02)	0.24	(0.02)
**Globus Pallidus**	**Left**	0.14	(0.01)	**0.12**	**(0.01)**	**0.12**	**(0.01)**	**0.12**	**(0.01)**	**0.11**	**(0.01)**	**0.11**	**(0.01)**
	**Right**	0.14	(0.01)	**0.12**	**(0.01)**	**0.13**	**(0.01)**	**0.12**	**(0.01)**	**0.12**	**(0.01)**	**0.12**	**(0.01)**
**Putamen**	**Left**	0.30	(0.02)	**0.24**	**(0.03)**	**0.26**	**(0.03)**	**0.27**	**(0.02)**	**0.26**	**(0.03)**	**0.25**	**(0.03)**
	**Right**	0.30	(0.02)	**0.26**	**(0.03)**	**0.28**	**(0.03)**	**0.27**	**(0.02)**	**0.27**	**(0.03)**	**0.28**	**(0.03)**
**Thalamus**	**Left**	0.37	(0.02)	**0.34**	**(0.02)**	0.36	(0.03)	0.38	(0.02)	0.36	(0.03)	**0.35**	**(0.03)**
	**Right**	0.36	(0.02)	0.35	(0.03)	0.37	(0.03)	0.37	(0.02)	0.36	(0.03)	0.37	(0.03)
**Cerebellum**		7.02	(0.66)	6.53	(0.82)	6.97	(0.31)	6.64	(0.40)	6.61	(0.84)	6.82	(0.37)

Supplementary Figure 1 Flow chart showing inclusion of PPA participants in the study. Abbreviations: *GRN*-NFV, nonfluent variant PPA due to progranulin mutation; *GRN*-NOS, not otherwise specified PPA due to a progranulin mutation; lvPPA, logopenic variant PPA; nfvPPA, nonfluent variant PPA; svPPA, semantic variant PPA.

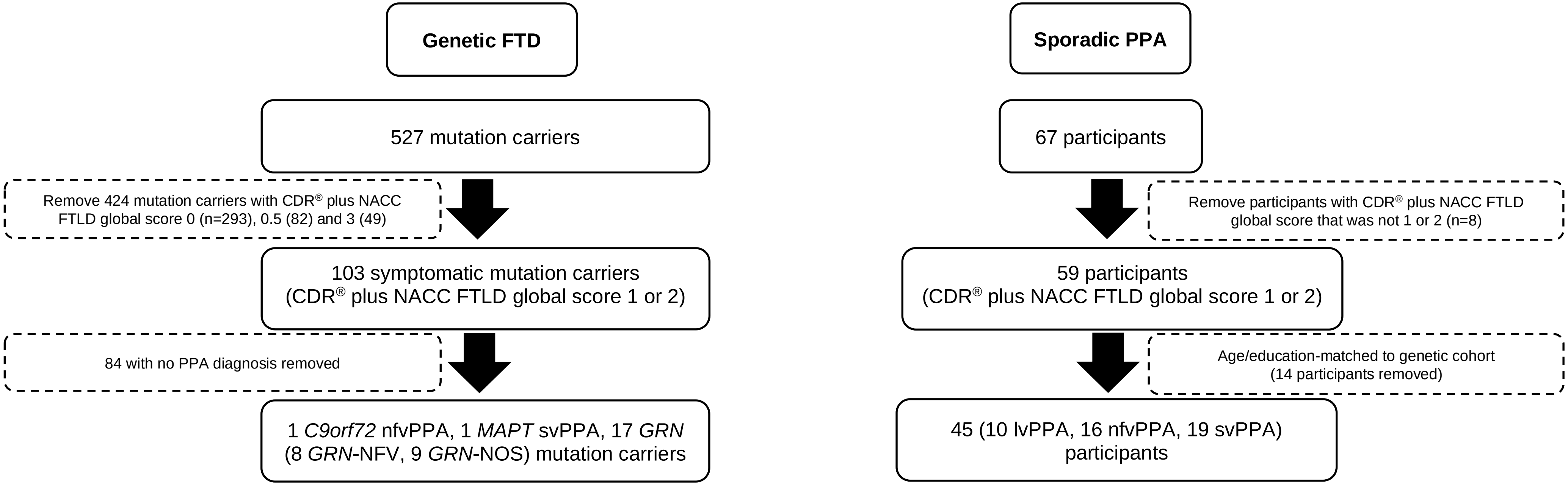

Supplementary Appendix. GENFI Consortium Affiliations

- Sónia Afonso - Instituto Ciencias Nucleares Aplicadas a Saude, Universidade de Coimbra, Coimbra, Portugal;
- Maria Rosario Almeida - Faculty of Medicine, University of Coimbra, Coimbra, Portugal;
- Sarah Anderl-Straub – Department of Neurology, University of Ulm, Ulm, Germany;
- Christin Andersson - Department of Clinical Neuroscience, Karolinska Institutet, Stockholm, Sweden;
- Anna Antonell - Alzheimer's disease and Other Cognitive Disorders Unit, Neurology Service, Hospital Clínic, Barcelona, Spain;
- Silvana Archetti - Biotechnology Laboratory, Department of Diagnostics, ASST Brescia Hospital, Brescia, Italy;
- Andrea Arighi - Fondazione IRCCS Ca' Granda Ospedale Maggiore Policlinico, Neurodegenerative Diseases Unit, Milan, Italy; University of Milan, Centro Dino Ferrari, Milan, Italy;
- Mircea Balasa - Alzheimer's disease and Other Cognitive Disorders Unit, Neurology Service, Hospital Clínic, Barcelona, Spain;
- Myriam Barandiaran - Cognitive Disorders Unit, Department of Neurology, Donostia University Hospital, San Sebastian, Gipuzkoa, Spain; Neuroscience Area, Biodonostia Health Research Insitute, San Sebastian, Gipuzkoa, Spain;
- Nuria Bargalló - Imaging Diagnostic Center, Hospital Clínic, Barcelona, Spain;
- Robart Bartha - Department of Medical Biophysics, The University of Western Ontario, London, Ontario, Canada; Centre for Functional and Metabolic Mapping, Robarts Research Institute, The University of Western Ontario, London, Ontario, Canada;
- Benjamin Bender - Department of Diagnostic and Interventional Neuroradiology, University of Tübingen, Tübingen, Germany;
- Alberto Benussi - Centre for Neurodegenerative Disorders, Department of Clinical and Experimental Sciences, University of Brescia, Italy;
- Maxime Bertoux – Inserm 1172, Lille, France; CHU, CNR-MAJ, Labex Distalz, LiCEND Lille, France;
- Anne Bertrand - Sorbonne Université, Paris Brain Institute – Institut du Cerveau – ICM, Inserm U1127, CNRS UMR 7225, AP-HP - Hôpital Pitié-Salpêtrière, Paris, France; Inria, Aramis project-team, F-75013, Paris, France; Centre pour l'Acquisition et le Traitement des Images, Institut du Cerveau et la Moelle, Paris, France
- Valentina Bessi - Department of Neuroscience, Psychology, Drug Research, and Child Health, University of Florence, Florence, Italy;
- Sandra Black - Sunnybrook Health Sciences Centre, Sunnybrook Research Institute, University of Toronto, Toronto, Canada;
- Sergi Borrego-Ecija - Alzheimer's disease and Other Cognitive Disorders Unit, Neurology Service, Hospital Clínic, Barcelona, Spain;
- Jose Bras - Center for Neurodegenerative Science, Van Andel Institute, Grand Rapids, Michigan, MI 49503, USA;

- Alexis Brice - Sorbonne Université, Paris Brain Institute – Institut du Cerveau – ICM, Inserm U1127, CNRS UMR 7225, AP-HP - Hôpital Pitié-Salpêtrière, Paris, France; Reference Network for Rare Neurological Diseases (ERN-RND);
- Rose Bruffaerts - Laboratory for Cognitive Neurology, Department of Neurosciences, KU Leuven, Leuven, Belgium;
- Agnès Camuzat - Sorbonne Université, Paris Brain Institute – Institut du Cerveau – ICM, Inserm U1127, CNRS UMR 7225, AP-HP - Hôpital Pitié-Salpêtrière, Paris, France;
- Marta Cañada - CITA Alzheimer, San Sebastian, Gipuzkoa, Spain
- Valentina Cantoni - Centre for Neurodegenerative Disorders, Neurology Unit, Department of Clinical and Experimental Sciences, University of Brescia, Brescia, Italy;
- Paola Caroppo - Fondazione IRCCS Istituto Neurologico Carlo Besta, Milano, Italy;
- Miguel Castelo-Branco - Faculty of Medicine, University of Coimbra, Coimbra, Portugal;
- Olivier Colliot - Sorbonne Université, Paris Brain Institute – Institut du Cerveau – ICM, Inserm U1127, CNRS UMR 7225, AP-HP - Hôpital Pitié-Salpêtrière, Paris, France; Inria, Aramis project-team, F-75013, Paris, France; Centre pour l'Acquisition et le Traitement des Images, Institut du Cerveau et la Moelle, Paris, France;
- Thomas Cope – Department of Clinical Neuroscience, University of Cambridge, Cambridge, UK;
- Vincent Deramecourt - Univ Lille, France; Inserm 1172, Lille, France; CHU, CNR-MAJ, Labex Distalz, LiCEND Lille, France;
- María de Arriba - Neuroscience Area, Biodonostia Health Research Insitute, San Sebastian, Gipuzkoa, Spain;
- Giuseppe Di Fede - Fondazione IRCCS Istituto Neurologico Carlo Besta, Milano, Italy;
- Alina Díez - Neuroscience Area, Biodonostia Health Research Insitute, San Sebastian, Gipuzkoa, Spain
- Diana Duro - Faculty of Medicine, University of Coimbra, Coimbra, Portugal;
- Chiara Fenoglio - Fondazione IRCCS Ca' Granda Ospedale Maggiore Policlinico, Neurodegenerative Diseases Unit, Milan, Italy; University of Milan, Centro Dino Ferrari, Milan, Italy;
- Camilla Ferrari - Department of Neuroscience, Psychology, Drug Research, and Child Health, University of Florence, Florence, Italy;
- Catarina B. Ferreira -Laboratory of Neurosciences, Institute of Molecular Medicine, Faculty of Medicine, University of Lisbon, Lisbon, Portugal;
- Nick Fox – Dementia Research Centre, Department of Neurodegenerative Disease, UCL Institute of Neurology, Queen Square, London, UK;
- Morris Freedman - Baycrest Health Sciences, Rotman Research Institute, University of Toronto, Toronto, Canada;
- Giorgio Fumagalli - Fondazione IRCCS Ca' Granda Ospedale Maggiore Policlinico, Neurodegenerative Diseases Unit, Milan, Italy; University of Milan, Centro Dino Ferrari, Milan, Italy;
- Aurélie Funkiewiez - Centre de référence des démences rares ou précoces, IM2A, Département de Neurologie, AP-HP - Hôpital Pitié-Salpêtrière, Paris, France; Sorbonne Université, Paris Brain Institute

– Institut du Cerveau – ICM, Inserm U1127, CNRS UMR 7225, AP-HP - Hôpital Pitié-Salpêtrière, Paris, France;

- Alazne Gabilondo -Neuroscience Area, Biodonostia Health Research Insitute, San Sebastian, Gipuzkoa, Spain;
- Roberto Gasparotti - Neuroradiology Unit, University of Brescia, Brescia, Italy
- Serge Gauthier - Alzheimer Disease Research Unit, McGill Centre for Studies in Aging, Department of Neurology & Neurosurgery, McGill University, Montreal, Québec, Canada;
- Stefano Gazzina - Neurology, ASST Brescia Hospital, Brescia, Italy
- Giorgio Giaccone - Fondazione IRCCS Istituto Neurologico Carlo Besta, Milano, Italy;
- Ana Gorostidi - Neuroscience Area, Biodonostia Health Research Insitute, San Sebastian, Gipuzkoa, Spain;
- Rita Guerreiro - Center for Neurodegenerative Science, Van Andel Institute, Grand Rapids, Michigan, MI 49503, USA;
- Carolin Heller – Dementia Research Centre, Department of Neurodegenerative Disease, UCL Institute of Neurology, Queen Square, London, UK;
- Tobias Hoegen - Neurologische Klinik, Ludwig-Maximilians-Universität München, Munich, Germany;
- Begoña Indakoetxea - Cognitive Disorders Unit, Department of Neurology, Donostia University Hospital, San Sebastian, Gipuzkoa, Spain; Neuroscience Area, Biodonostia Health Research Insitute, San Sebastian, Gipuzkoa, Spain;
- Vesna Jelic - Division of Clinical Geriatrics, Karolinska Institutet, Stockholm, Sweden;
- Hans-Otto Karnath - Division of Neuropsychology, Hertie-Institute for Clinical Brain Research and Center of Neurology, University of Tübingen, Tübingen, Germany;
- Ron Keren -The University Health Network, Toronto Rehabilitation Institute, Toronto, Canada;
- Gregory Kuchcinski - Univ Lille, France; Inserm 1172, Lille, France; CHU, CNR-MAJ, Labex Distalz, LiCEND Lille, France;
- Tobias Langheinrich - Division of Neuroscience and Experimental Psychology, Wolfson Molecular Imaging Centre, University of Manchester, Manchester, UK; Manchester Centre for Clinical Neurosciences, Department of Neurology, Salford Royal NHS Foundation Trust, Manchester, UK;
- Thibaud Lebouvier - Univ Lille, France; Inserm 1172, Lille, France; CHU, CNR-MAJ, Labex Distalz, LiCEND Lille, France;
- Maria João Leitão - Centre of Neurosciences and Cell Biology, Universidade de Coimbra, Coimbra, Portugal;
- Albert Lladó - Alzheimer’s disease and Other Cognitive Disorders Unit, Neurology Service, Hospital Clínic, Barcelona, Spain;
- Gemma Lombardi - Department of Neuroscience, Psychology, Drug Research and Child Health, University of Florence, Florence, Italy;
- Sandra Loosli -Neurologische Klinik, Ludwig-Maximilians-Universität München, Munich, Germany;
- Carolina Maruta - Laboratory of Language Research, Centro de Estudos Egas Moniz, Faculty of Medicine, University of Lisbon, Lisbon, Portugal;

- Simon Mead - MRC Prion Unit, Department of Neurodegenerative Disease, UCL Institute of Neurology, Queen Square, London, UK;
- Lieke Meeter - Department of Neurology, Erasmus Medical Center, Rotterdam, Netherlands;
- Gabriel Miltenberger - Faculty of Medicine, University of Lisbon, Lisbon, Portugal;
- Rick van Minkelen - Department of Clinical Genetics, Erasmus Medical Center, Rotterdam, Netherlands;
- Sara Mitchell - Sunnybrook Health Sciences Centre, Sunnybrook Research Institute, University of Toronto, Toronto, Canada;
- Katrina Moore – Dementia Research Centre, Department of Neurodegenerative Disease, UCL Institute of Neurology, Queen Square, London UK;
- Benedetta Nacmias - Department of Neuroscience, Psychology, Drug Research and Child Health, University of Florence, Florence, Italy;
- Annabel Nelson - Dementia Research Centre, Department of Neurodegenerative Disease, UCL Institute of Neurology, Queen Square, London, UK;
- Linn Öijerstedt - Center for Alzheimer Research, Division of Neurogeriatrics, Department of Neurobiology, Care Sciences and Society, Bioclinicum, Karolinska Institutet, Solna, Sweden; Unit for Hereditary Dementias, Theme Aging, Karolinska University Hospital, Solna, Sweden;
- Jaume Olives - Alzheimer's disease and Other Cognitive Disorders Unit, Neurology Service, Hospital Clínic, Barcelona, Spain;
- Sebastien Ourselin - School of Biomedical Engineering & Imaging Sciences, King's College London, London, UK;
- Alessandro Padovani - Centre for Neurodegenerative Disorders, Department of Clinical and Experimental Sciences, University of Brescia, Italy
- Jessica Panman – Department of Neurology, Erasmus Medical Center, Rotterdam, Netherlands;
- Janne M. Papma - Department of Neurology, Erasmus Medical Center, Rotterdam;
- Yolande Pijnenburg - Amsterdam University Medical Centre, Amsterdam VUmc, Amsterdam, Netherlands;
- Cristina Polito - Department of Biomedical, Experimental and Clinical Sciences "Mario Serio", Nuclear Medicine Unit, University of Florence, Florence, Italy
- Enrico Premi - Stroke Unit, ASST Brescia Hospital, Brescia, Italy
- Sara Prioni - Fondazione IRCCS Istituto Neurologico Carlo Besta, Milano, Italy;
- Catharina Prix -Neurologische Klinik, Ludwig-Maximilians-Universität München, Munich, Germany;
- Rosa Rademakers [as London Ontario geneticist] - Department of Neurosciences, Mayo Clinic, Jacksonville, Florida, USA;
- Veronica Redaelli -Fondazione IRCCS Istituto Neurologico Carlo Besta, Milano, Italy;
- Daisy Rinaldi - Centre de référence des démences rares ou précoces, IM2A, Département de Neurologie, AP-HP - Hôpital Pitié-Salpêtrière, Paris, France; Sorbonne Université, Paris Brain Institute – Institut du Cerveau – ICM, Inserm U1127, CNRS UMR 7225, AP-HP - Hôpital Pitié-Salpêtrière, Paris,

France; Département de Neurologie, AP-HP - Hôpital Pitié-Salpêtrière, Paris, France; Reference Network for Rare Neurological Diseases (ERN-RND);

- Tim Rittman – Department of Clinical Neurosciences, University of Cambridge, Cambridge, UK;
- Ekaterina Rogaeva -Tanz Centre for Research in Neurodegenerative Diseases, University of Toronto, Toronto, Canada;
- Adeline Rollin - CHU, CNR-MAJ, Labex Distalz, LiCEND Lille, France;
- Pedro Rosa-Neto -Translational Neuroimaging Laboratory, McGill Centre for Studies in Aging, McGill University, Montreal, Québec, Canada;
- Giacomina Rossi - Fondazione IRCCS Istituto Neurologico Carlo Besta, Milano, Italy;
- Martin Rossor – Dementia Research Centre, Department of Neurodegenerative Disease, UCL Institute of Neurology, Queen Square, London, UK;
- Beatriz Santiago - Neurology Department, Centro Hospitalar e Universitario de Coimbra, Coimbra, Portugal;
- Dario Saracino – Sorbonne Université, Paris Brain Institute – Institut du Cerveau – ICM, Inserm U1127, CNRS UMR 7225, AP-HP - Hôpital Pitié-Salpêtrière, Paris, France; Inria, Aramis project-team, F-75013, Paris, France; Centre de référence des démences rares ou précoces, IM2A, Département de Neurologie, AP-HP - Hôpital Pitié-Salpêtrière, Paris, France;
- Sabrina Sayah - Sorbonne Université, Paris Brain Institute – Institut du Cerveau – ICM, Inserm U1127, CNRS UMR 7225, AP-HP - Hôpital Pitié-Salpêtrière, Paris, France;
- Elio Scarpini - Fondazione IRCCS Ca’ Granda Ospedale Maggiore Policlinico, Neurodegenerative Diseases Unit, Milan, Italy; University of Milan, Centro Dino Ferrari, Milan, Italy;
- Sonja Schönecker - Neurologische Klinik, Ludwig-Maximilians-Universität München, Munich, Germany;
- Elisa Semler -Department of Neurology, University of Ulm, Ulm;
- Rachelle Shafei – Dementia Research Centre, Department of Neurodegenerative Disease, UCL Institute of Neurology, Queen Square, London, UK;
- Christen Shoesmith - Department of Clinical Neurological Sciences, University of Western Ontario, London, Ontario, Canada;
- Imogen Swift - Department of Neurodegenerative Disease, Dementia Research Centre, UCL Institute of Neurology, Queen Square, London, UK;
- Miguel Tábuas-Pereira - Neurology Department, Centro Hospitalar e Universitario de Coimbra, Coimbra, Portugal;
- Mikel Tainta - Neuroscience Area, Biodonostia Health Research Insitute, San Sebastian, Gipuzkoa, Spain;
- Ricardo Taipa - Neuropathology Unit and Department of Neurology, Centro Hospitalar do Porto - Hospital de Santo António, Oporto, Portugal;
- David Tang-Wai -The University Health Network, Krembil Research Institute, Toronto, Canada;
- David L Thomas - Neuroimaging Analysis Centre, Department of Brain Repair and Rehabilitation, UCL Institute of Neurology, Queen Square, London, UK;

- Paul Thompson - Division of Neuroscience and Experimental Psychology, Wolfson Molecular Imaging Centre, University of Manchester, Manchester, UK;
- Hakan Thonberg - Center for Alzheimer Research, Division of Neurogeriatrics, Karolinska Institutet, Stockholm, Sweden;
- Carolyn Timberlake – Department of Clinical Neurosciences, University of Cambridge, Cambridge, UK;
- Pietro Tiraboschi - Fondazione IRCCS Istituto Neurologico Carlo Besta, Milano, Italy;
- Emily Todd - Dementia Research Centre, Department of Neurodegenerative Disease, UCL Institute of Neurology, UK;
- Philip Van Damme - Neurology Service, University Hospitals Leuven, Belgium; Laboratory for Neurobiology, VIB-KU Leuven Centre for Brain Research, Leuven, Belgium;
- Mathieu Vandenbulcke - Geriatric Psychiatry Service, University Hospitals Leuven, Belgium; Neuropsychiatry, Department of Neurosciences, KU Leuven, Leuven, Belgium;
- Michele Veldsman - Nuffield Department of Clinical Neurosciences, Medical Sciences Division, University of Oxford, Oxford, UK;
- Ana Verdelho - Department of Neurosciences and Mental Health, Centro Hospitalar Lisboa Norte - Hospital de Santa Maria & Faculty of Medicine, University of Lisbon, Lisbon, Portugal;
- Jorge Villanua - OSATEK, University of Donostia, San Sebastian, Gipuzkoa, Spain;
- Carlo Wilke - Department of Neurodegenerative Diseases, Hertie-Institute for Clinical Brain Research and Center of Neurology, University of Tübingen, Tübingen, Germany; Center for Neurodegenerative Diseases (DZNE), Tübingen, Germany;
- Ione Woollacott – Dementia Research Centre, Department of Neurodegenerative Disease, UCL Institute of Neurology, Queen Square, London, UK;
- Elisabeth Wlasich - Neurologische Klinik, Ludwig-Maximilians-Universität München, Munich, Germany;
- Henrik Zetterberg - Dementia Research Institute, Department of Neurodegenerative Disease, UCL Institute of Neurology, Queen Square, London, UK;
- Miren Zulaica - Neuroscience Area, Biodonostia Health Research Insitute, San Sebastian, Gipuzkoa, Spain.
